# Tranexamic acid impairs plasmin generation on human mesenchymal stem cells and derived membrane microvesicles, halting pericellular proteolysis

**DOI:** 10.3389/fmed.2025.1570395

**Published:** 2025-06-30

**Authors:** Ramy Abou Rjeily, Christina Mrad, Fatiha Z. El-Ghazouani, Florence Toti, Audrey Cras, Eduardo Angles-Cano

**Affiliations:** ^1^Université Paris Cité, INSERM, Optimisation Thérapeutique en Neuropharmacologie U1144, Paris, France; ^2^INSERM (French National Institute of Health and Medical Research), UMR 1260, Regenerative Nanomedicine, University of Strasbourg, Strasbourg, France; ^3^AP-HP, Hôpital Saint-Louis, Unité de Thérapie Cellulaire, Centre d’Investigation Clinique de Biothérapies CBT501, Paris, France; ^4^Université Paris Cité, INSERM UMR 1342, Paris, France

**Keywords:** pericellular proteolysis, urokinase plasminogen activator receptor, plasminogen activation pathway, membrane microvesicles, tranexamic acid, mesenchymal stem cells, urokinase

## Abstract

**Introduction:**

Mesenchymal stem cells (MSCs) participate in the dynamic remodeling of the extracellular matrix during wound healing, natural bleeding processes or cancer progression. Pericellular proteolysis is a key mechanism mediating the aforementioned processes.

**Aim:**

This study primarily aimed to define mechanistic pathways of plasmin formation and its consequences on MSC phenotype and functioning. We have also investigated the regulatory mechanisms mediated by PAI-1 and the ability of MSCs to shed microvesicles bearing the proteolytic machinery.

**Methods:**

Human MSCs were derived from bone marrow or umbilical cord donors. Cells thus obtained were seeded in multi-well plates and treated with different concentrations of plasminogen and pro-urokinase in the presence or absence of variable amounts of tranexamic acid. We measured MVs formation and phenotypical changes occurring on MSCs. The amount of plasmin formed was quantified by western blot along with the plasmin activity detected by photometry.

**Results:**

We demonstrate that vesiculation is the early response of plasmin formation at the membrane of MSCs followed by cell retraction and detachment. We measured the effect of TXA on plasmin formation and its consequences on cell behavior. Our findings provide the first demonstration that TXA efficiently inhibits MSC-driven plasmin generation by competitively blocking plasminogen binding to the uPA•uPAR complex at the cell plasma membrane.

**Discussion:**

We propose that plasmin formation on MSCs may be involved in pathological processes such as endometrial hemorrhage (metrorrhagia and Post-Partum Hemorrhage), autoimmune and ischaemic diseases, as well as cancer. By advancing our understanding of these mechanisms, we open new avenues for the development of biomarkers and targeted treatments.

## Introduction

Pericellular proteolysis and fibrinolysis depend on plasmin formation at the cell membrane, the extracellular matrix or fibrin. For instance, plasmin formed by tissue plasminogen activator (tPA) from plasminogen bound to fibrin promotes fibrinolysis and thrombus lysis. In contrast, plasmin formed on the cell membrane by urokinase plasminogen activator (uPA) cleaves specific membrane-associated proteins that stimulate signalization leading to secretion of other mediators and microvesiculation that may support paracrine effects. The proteolytic action of membrane-bound plasmin on the surrounding extracellular matrix components promotes cell migration but may also impact wound healing under certain conditions (depending on the balance of proteolytic activity). Plasmin has many other physiologically significant substrates, including pro-metalloproteinases and cytokines. Of particular interest within this proteolytic context are human mesenchymal stem cells (MSCs) that migrate from the bone marrow into the circulation toward damaged tissues thus allowing tissue repair (1). In agreement with this mechanism, previous studies have identified in MSC cultures mRNA for uPA, its receptor (uPAR) and plasminogen activator inhibitor 1 (PAI-1) as part of their transcriptoma (2–4). Thus, it has recently been shown that uPA may act as a chemoattractant factor (5) and that uPAR activates signaling pathways facilitating the adhesion, migration, proliferation and differentiation of MSCs. These uPAR-initiated signaling cellular effects have been recently reviewed by Alfano et al. (6). Indirect *in vitro* data indicate that the uPA-uPAR plasminogen activation system of MSCs may participate in their migration into a clot (2, 7). Thus, the uPAR is a multifunctional receptor that besides signal transduction may also participate in surface-associated pericellular proteolysis. However, data linking cell phenotype and microvesiculation to plasmin generation or its potential regulation by inhibitors of either uPA or plasminogen binding to their cell membrane are scarce. From the more than 12 glycoproteins that function as plasminogen receptors, a few has been identified in MSC of different origin—α-enolase in human adipose tissue-derived MSC and in the mesothelial cell surface together with Annexin A2, and Plg-RKT- Plg receptors (8–10). In particular, tranexamic acid (TXA) that has the ability to inhibit plasminogen binding to membrane glycoproteins, has not been investigated as yet in MSCs proteolytic activity. A study on the effect of TXA on plasminogen binding to these receptors is therefore awaiting. On the other hand, inhibition of plasmin and uPA by, respectively, α_2_-antiplasmin and PAI-1, is a widely accepted model for the regulation of the uPA-uPAR system. However, there is currently no consensus on the mechanism of MSC detachment and survival via plasminogen activation or PAI-1 anti-adhesive properties (3).

Pericellular proteolysis and cell intercommunication play critical roles in the migration and implantation of MSCs in injured-ischemic tissues and wound healing. This study primarily aimed to define the functional assembly of plasminogen activation system components on MSCs and its regulation by TXA. Additionally, we have investigated the ability of MSCs to shed macrovesicles and transfer intercellular information and other regulatory mechanisms mediated by PAI-1. These different mechanisms are potentially relevant to clinical applications in haemorrhagic and ischaemic diseases and to the development of biomarkers for diagnostic purposes, shedding the light on potential therapeutic prevention of plasminogen-dependent pericellular proteolysis using TXA.

## Materials and methods

### Reagents and proteins

*Purified human proteins.* Glu-plasminogen was purified and characterized as described (11). Plasmin was prepared by activation of Glu-plasminogen with immobilized uPA according to Wiman and Wallen (12) with modifications. Recombinant plasminogen activator inhibitor 1 (rPAI-1) was kindly provided by Prof. Paul Declerck, (Faculty of Pharmaceutical Sciences, Katholieke Universiteit of Leuven, Belgium). Recombinant single-chain urokinase-type plasminogen activator (scuPA) was provided by Carlo Erba (Milan, Italy). Two-chain urokinase (uPA) was obtained from Prospec® (Tebu-Bio, France). α-thrombin (3,000 NIH units/mg) was from Enzyme Research Laboratories (Swamsea, UK). Basic fibroblast growth factor (bFGF) was from R&D Systems (Lille, France). Aprotinin (Trasylol®) was a gift from Bayer (Leverkusen, Germany). Carboxypeptidase B (CpB, porcine pancreas), the lysine analogue *trans*-4-(Aminomethyl)cyclohexanecarboxylic (tranexamic acid, TXA) acid and the tetrazolium salt 3-[4,5-dimethylthiazol-2-yl]-2,5-diphenyl tetrazolium bromide (MTT), were from Sigma-Aldrich (Saint-Louis, MO, USA). Exacyl® from Cheplapharm, France. Bovine serum albumin (Fraction V, protease-free) was from Eurobio (Courtaboeuf, France). L-glutamine, GlutaMAX-1, fetal bovine serum (FBS), DMEM-Ham’s F-12, minimum essential medium-α (MEMα), RPMI-1640, Hank’s balanced salt solution containing Ca^2+^ and Mg^2+^ (HBSS-CaMg), Dulbecco’s phosphate-buffered saline (PBS), Ficoll-Paque, penicillin–streptomycin solution were from Life Technologies (Cergy-Pontoise, France). The chromogenic substrate CBS0065 (methylmalonyl)-hydroxyprolylarginine-*para*-nitroanilide selective for plasmin was kindly provided by G. Contant (Stago, Gennevilliers, France). *Monoclonal antibodies.* The peroxidase-labeled monoclonal antibody directed against plasminogen kringle 1 (CPL15-PO) was obtained as described (13, 14); Anti-human uPAR IgG1mAbs IID7 (D2 domain), R3 (D1 domain) and R4 (D2D3 domain) were provided by V. Magdolen (KliFo/TUM, München, Germany) (15) and G. Høyer-Hansen (The Finsen Laboratory, Copenhagen, Denmark), respectively (16, 17). *Polyclonal antibodies.* Sheep anti-human recombinant scuPA and control pre-immune IgG fractions were prepared as described (11); Anti-goat Dylight 800-labeled anti-mouse IgG and anti-sheep IgG were from Jackson ImmunoResearch (West Grove, PA, USA).

### Mesenchymal stem cells isolation and characterization

Human bone marrow samples from healthy donors (*n* = 4) were harvested from washed filters used during bone marrow graft processing for allogeneic bone marrow transplantation (Cell Therapy Unit, Saint Louis Hospital, AP-HP, France). All samples were obtained after a written and informed consent following the Helsinki’s declaration. The collection and preparation of human samples has been approved by French Authorities (number AC-2024-6640). Bone marrow human mesenchymal stem cells were isolated and expanded as described previously (18). Briefly, mononucleated cells obtained from bone marrow samples by gradient centrifugation with Ficoll-Paque were cultured in MEMα supplemented with 10% fetal bovine serum (FBS), 2 mM Glutamax-1, 1 ng/mL bFGF, and 1% antibiotics/antimycotic. After 24–48 h, non-adherent cells were removed and medium was changed. After 2 weeks, adherent cells were then detached with trypsin, washed and cultured by seeding at 5,000 cells/cm^2^. Cultures were fed every 2 or 3 days until confluence. MSCs can grow and proliferate *in vitro* as plastic-adherent cells during several passages. Umbilical cord Mesenchymal Stem Cell (UC-MSCs) from each umbilical cord (UC) donor were cultured and expanded for one to three passages as indicated above, to obtain a sufficient cell quantity for the preparation of a Master Cell Bank, comprising multiple batches derived from a single donor. For this study, UC-MSCs from four distinct donors were employed. Experimental reproducibility was assessed by culturing and analyzing multiple samples derived from the same donor.

Each experiment was independently conducted in triplicate and repeated a minimum of three times using freshly thawed and cultured cells. To maintain consistency across experimental conditions, cells were used up to passage 4 and seeded at 90% confluence.

Monoclonal antibodies conjugated with either fluorescein isothiocyanate or phycoerythrin and directed to CD34, CD45, CD73, CD90, CD105, or matched isotype control (all purchased from Becton Dickinson, Le Pont de Claix, France) were used for hMSC immunophenotyping according to the manufacturer’s protocol. Data were acquired and analyzed on a 12-parameter flow cytometer (Navios EX system, Beckman Coulter) with Navios Plateform System software (Beckman Coulter). In accordance with the definition of hMSCs established by the International Society for Cellular Therapy (19), the bone marrow hMSCs used in our study did not express hematopoietic antigens such as CD34 and CD45, and were found positive for CD73, CD90, and CD105 expression.

### Identification of MSC plasminogen activators

Human MSCs were expanded in 25 cm^2^ or 75 cm^2^ tissue culture flasks depending on experiments. Cells were grown in a 37°C humidified atmosphere of 5% CO_2_ using DMEM-Ham’s F-12 medium. Cells were then seeded in 96 or 24 well plates, depending on experiments. At day 5 after seeding, conditioned medium was collected, the monolayer of cells washed and then lysed in 100 mM Tris–HCl buffer, pH 8.1, containing 1% Triton X-100. Fibrin autography following SDS-PAGE was performed as described previously (20). Proteins in conditioned media and cell lysates (100 μg) and reference proteins (10 μL of tPA 5 nM, uPA 1 nM and plasmin 500 nM) were electrophoresed in an 8% polyacrylamide gel under non-reducing conditions. Fibrin autography following SDS-PAGE was performed on fibrin-plasminogen-agarose gels as previously described (21). Briefly, proteins in cell lysates (100 μg) and reference proteins (10 μL of tPA 5 nM, uPA 1 nM and plasmin 250 nM) were electrophoresed as indicated above. SDS was then exchanged with 2.5% Triton X-100. After washing-off excess Triton X-100 with distilled water, the gel was carefully overlaid on a 1% agarose gel containing 1 mg/mL of bovine fibrinogen, 100 nM plasminogen and 0.2 NIH unit/mL of bovine thrombin. Zymograms were allowed to develop at 37°C during 24 h and photographed at regular intervals using dark-ground illumination. Active proteins in cell lysates were identified by reference to the migration of known fibrinolytic agents uPA (*Mr* ~54,000), PAI-1 (~47,000), tPA (~71,000), plasmin (85,000). To verify the activator identity, the fibrin-agarose gels were supplemented with 10 μg/mL IgG polyclonal antibody directed against uPA.

### Detection of the urokinase plasminogen activator receptor (uPAR)

The presence of uPAR at the membrane of MSCs was detected and measured following two approaches: binding of scuPA to cells and immunoblotting of MSC-derived membrane microvesicles (MVs).

*Binding of scuPA to cells.* Increasing concentrations (0–100 nM) of scuPA, in HBSS buffer containing 4 mg/mL BSA were incubated during 1 h at 37°C with cultured cells in a humidified atmosphere of 5% CO_2_. Unbound scuPA was then eliminated and cells were washed twice with HBSS. To verify the specificity of the binding, a fixed concentration of native scuPA (0.1 nM) was mixed with a 10 molar excess (1 nM) of a recombinant inactive form “r-scuPA Ile 1593Gly” (kindly provided by H.R. Lijnen, University of Leuven, Leuven, Belgium). The native scuPA specifically bound to cells was detected by measuring its capacity to activate plasminogen (1 μM) in the presence of the chromogenic substrate CBS0065 (0.75 mM). Rates of plasmin formation were calculated from slopes of A_405nm/_A_490nm_ *versus* time. Initial rates versus uPAR concentration data were fitted according to the Langmuir equation for single site interactions using KaleidaGraph v. 4.5.

*Immunoblotting of membrane-bound uPAR.* To detect uPAR anchored to the membrane of MSCs, MSC-derived MVs were lysed in 100 mM Tris–HCl buffer, pH 8.1, containing 1% Triton X-100. Proteins in lysates (10 μg) were electrophoresed in an 8% polyacrylamide gel under reducing conditions. Proteins were transferred to PVDF membranes and revealed with primary specific monoclonal antibodies anti-human uPAR IgG1mAbs IID7, R3 and R4 kindly provided by V. Magdolen (KliFo/TUM, München, Germany) and G. Høyer-Hansen (The Finsen Laboratory, Copenhagen, Denmark) at proper concentrations. Anti-goat Dylight 800 Dylight 800-labeled anti-mouse IgG and anti-sheep IgG were from Jackson ImmunoResearch (West Grove, PA, USA).

### MSC activation by plasmin generated at their membrane

*Activity assay.* Human MSCs seeded in 96-well plates were washed with HBSS buffer containing 4 mg/mL BSA (HBSS-BSA) and then incubated with HBSS-BSA containing 5 nM scuPA for 1 h at 37°C. Unbound scuPA was eliminated by washing cells twice with HBSS and varying concentrations of plasminogen (0–2 μM) and 0.75 mM of CBS0065 a plasmin selective chromogenic substrate, were added. Kinetics of plasmin formation was followed during 4 h by measuring the release of *p*-nitroaniline from the chromogenic substrate, detected as a change in absorbance (ΔA_405nm_/A_490nm_ min), using a multi-well plate reader (MX5000, Dynex) thermostated at 37°C. Rates of plasmin formation were calculated from slopes of A_405nm_/A_490nm_ *versus* time. Initial rates were computer-calculated at the inflexion point of the curve by non-linear least square fitting.

In parallel experiments, the effect of CpB, an exopeptidase that cleaves C-ter Lys residues of membrane proteins, and aprotinin, a serine-protease inhibitor with restricted specificity, on the activation of plasminogen (1 μM) was determined (data not shown).

*Immunoblotting*. To detect plasminogen cleavage and plasmin formation at the cell membrane, cell lysates and supernatants from plasminogen activation experiments were analyzed by Western blotting using CPL15 HRP-labeled mAb specific for plasminogen kringle 1 (see Supplementary Figure 1 for plasminogen structure). Cleavage of 1 μM plasminogen and plasmin formation was detected at different time points. Samples were electrophoresed in 8% polyacrylamide gel under reducing conditions. Proteins were transferred to PVDF membranes and revealed with the anti-plasminogen kringle 1 CPL15-HRP mAb by chemiluminescence (ECL+, Amersham Biosciences, Little Chalfont, UK).

### Effects of membrane-bound plasmin on cell phenotype and survival

Human MSCs seeded in 96-well plates washed with HBSS-BSA buffer were incubated with plasminogen at 1 μM under culture conditions and their morphological phenotype (retraction of the cell monolayer in response to plasmin formation) was evaluated by microphotography at different time points. The following analyses were performed after the plasminogen activation experiments. To quantify the extent of detachment, non-adherent cells were eliminated by two washes in PBS and residual adherent cells were stained with 0.5 mg/mL of MTT. After 1 h at 37°C, excess MTT was removed and the formazan crystals were dissolved in 100 μL of DMSO and detected at A_550nm_. Absorbance readings are proportional to the number of living cells.

### Isolation and quantification of microvesicles

Microvesicles were isolated from MSCs conditioned medium cultured for 24–48 h at 37°C in a humidified atmosphere of 5% CO_2_. Culture supernatants were collected and cleared from detached cells or large cell fragments by double centrifugation at 1,500*g* for 15 min followed by 13,000*g* for 3 min at 4°C. The supernatants were then centrifuged at 20,000*g* for 90 min at 4°C. Pelleted MVs were washed twice using the same conditions and were suspended in PBS in a final volume of 100 μL.

The amount of MVs was quantitated by measuring protein concentration at A_280nm_ in MVs lysates produced by thermic shock (5 cycles at 37°C and −196°C) using a NanoDrop® ND-1000 spectrophotometer (Labtech, Palaiseau, France). Total MVs concentration was also determined by taking advantage of the MVs ubiquitous phosphatidylserine (Phtdser) exposure (22). MVs were captured onto insolubilized biotinylated annexin-5, a protein with high affinity for Phtdser, using streptavidin-coated microtitration plates (Roche Diagnostics, Meylan, France). After 3 washes, MVs were measured by prothrombinase assay in which blood clotting factors (FXa, FVa, FII) and calcium concentrations were added to ensure that the MVs Phtdser is the rate-limiting parameter of the reaction. Data are expressed either as mg/ml of MV protein content by reference to a calibration curve constructed with purified albumin or as nM PhtdSer equivalents by reference to a standard curve constructed with liposomes of known PhtdSer content (22).

### Plasminogen activator inhibitor 1

*Release of PAI-1 by MSC.* Supernatants (*n* = 96) obtained from MSC cultures (24–48 h) were used to quantify PAI-1 activity by titration against uPA as follow. Equal volumes of MSC supernatant and 5 iu/mL (0.926 nM) uPA were incubated for 15 min at room temperature. This time was sufficient to ensure inhibition of uPA activity by PAI-1 present in the supernatant. Residual uPA activity was then measured by adding an equal volume of assay buffer (composed of Sodium phosphate 0.05 mole/L, NaCl 0.08 mole/L and Azide 0.01%) containing 1 μM plasminogen and 0.75 mM of the plasmin selective chromogenic substrate CBS0065. Cleavage of CBS0065 by active plasmin was monitored by photometry at A405 nm. The residual uPA activity was calculated using a standard curve constructed using different concentrations of uPA as reference. The amount of uPA inhibited is equivalent to the quantity of PAI-1 present in the supernatant. Briefly, the concentration of PAI-1 (in IU/mL) was converted to ng/mL based on the known specific activity of uPA (100,000 IU/mg; 1 IU = 10 ng) and the molecular masses of uPA (~54 kDa) and PAI-1 (~47 kDa). Since PAI-1 inhibits uPA in a 1:1 molar ratio, the ng/mL concentration of PAI-1 was calculated by multiplying the determined IU/mL value by the molecular weight ratio adjustment: PAI-1 (ng/mL) = PAI-1 (IU/mL) × 10 × 54/47. The concentration is appropriately adjusted for molecular weight differences while maintaining the 1:1 stoichiometry of uPA-PAI-1 inhibition.

### The uPA bound to uPAR is protected from inhibition by PAI-1

To investigate the role of PAI-1 on plasmin generation at the cell membrane, we compared the effect of PAI-1 on cell-bound uPA versus uPA added to the microenvironment of the cell. scuPA known to be resistant to PAI-1 inhibition, was used as a control (see Supplementary Figures 2, 3). The activity of both scuPA and uPA previously bound to the cell membrane was not sensitive to the activity of PAI-1 present in the supernatant. In contrast, uPA but not scuPA added to the MSC-conditioned medium, was efficiently inhibited by the PAI-1 secreted by MSCs.

The limited decrease in bound uPA activity can be attributed to the release of small amounts of uPA from uPAR allowing its inhibition by PAI-1. The interaction of uPA with uPAR although of high affinity, is reversible and it may therefore be released from the membrane. Indeed, uPA•PAI-1 complexes were detected in the supernatant by reverse fibrin zymography.

### Exogenous regulation of pericellular proteolysis by TXA

Tranexamic acid, a lysine analogue, impairs plasminogen activation by blocking lysine-binding sites (LBS) on plasminogen kringle 1 and 4. Given its role as a prototype interventional regulator of disordered fibrinolysis, we investigated TXA’s capacity to modulate plasmin generation on mesenchymal stem cells. To determine the inhibition constant (Ki) of TXA for plasminogen binding to MSC membrane glycoproteins, plasminogen (200, 300, and 400 nM) was pre-incubated with varying TXA concentrations for 15 min at 4°C. The mixture was then supplemented with the plasmin selective chromogenic substrate CBS0065, and 100 μL of this solution was added to MSC-seeded microplate wells pre-treated with 5 nM uPA. Plasmin generation kinetics were monitored at 37°C for 2 h using a 96-well plate reader (MX5000, Dynex). To assess TXA’s effect on MSC behavior, cell morphology changes (retraction and detachment) were observed using a ZEISS AXIO OBSERVER microscope at 40× magnification. Images were captured using a charge-coupled device camera (QIclick FCLR-12, Qimaging, Roper Scientific). For MSC viability, the MTT assay was performed. At indicated time points during plasminogen activation, conditioned medium was carefully removed and replaced with 100 μL of 0.5 mg/mL MTT solution. After 1 h at 37°C, excess MTT was removed, and formazan crystals were solubilized in 100 μL of DMSO. Absorbance at 550 nm was measured, with readings proportional to the number of living cells. Results were expressed as a percentage relative to untreated controls. Similar experiments were conducted with MSC-derived microvesicles to evaluate TXA’s impact on MVs plasmin generation and intercellular communication.

### Statistical analysis

For our study we used MSC obtained from 4 different umbilical cord or bone marrow donors. Reproducibility between different samples frozen from the same donor was confirmed in preliminary experiments. All experiments were performed in triplicate for each condition and at least three times for each batch of freshly thawed and cultured cells. Values from the enzymatic assays were calculated by reference to a standard curve that ensured inter-assay consistency (Interassay variations of computed data never exceeded 10%). Data from all experiments are presented as mean ± standard deviation. Unless otherwise stated, the statistical analysis was performed using 2 tailed Student’s t-test or 1-way or 2-way ANOVA.

## Results

### Identification of uPA and PAI-1 expressed by MSCs

**Figure 1:**
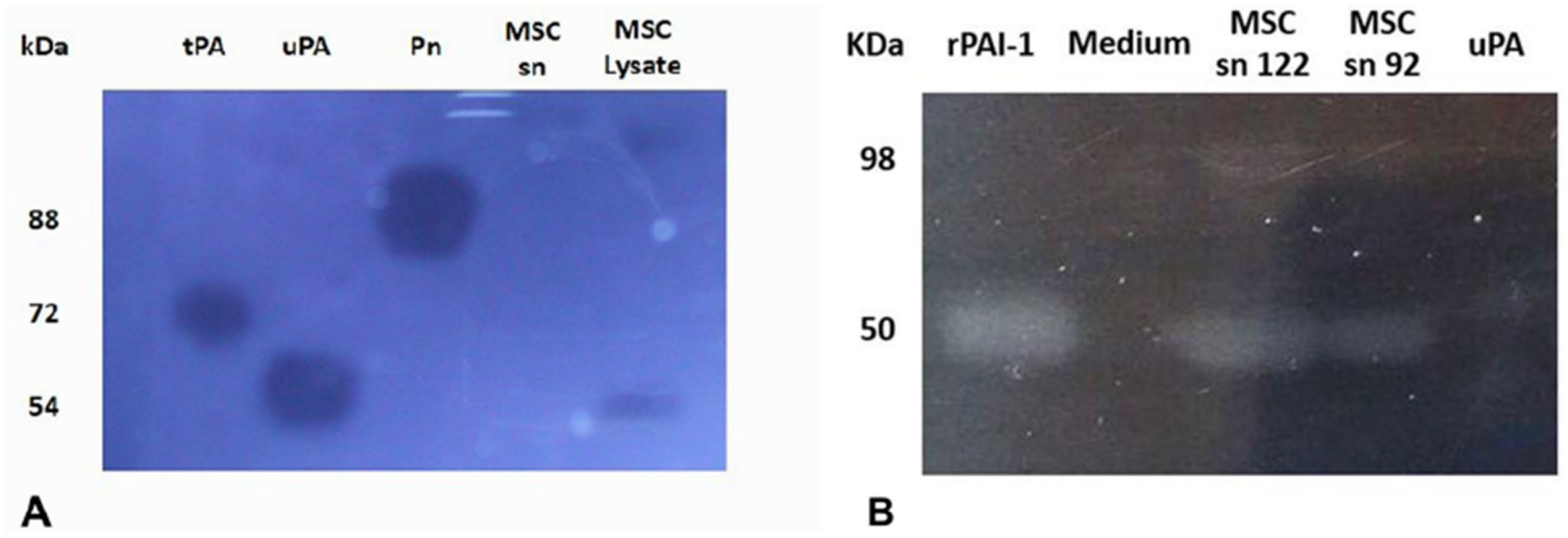
Identification of plasminogen activators and plasminogen activator inhibitor-1 by zymographic analysis. The samples were electrophoresed in SDS-PAGE gels. The SDS was exchanged by Triton X100 to restore protein structure and function. The Triton X100 gel was overlaid on fibrin-agar plate-gels to develop protein activities. MSCs are shown to express active uPA **(A)** and active PAI-1 **(B)**, identified on the basis of their molecular mobility compared to references shown at the left. The picture was taken after 24 h at 37°C using dark-ground illumination. The position of purified control protein references is indicated on the left. **(A)** *Fibrin-agarose zymography of MSC lysate*. MSCs are shown to express active urokinase plasminogen activator (uPA), identified on the basis of its molecular mobility compared to references. The Triton X100 gel was overlaid on plasminogen-fibrin-agar plate-gels to develop plasminogen activator activities. kDa (molecular weight position of protein markers in the SDS-PAGE gel). tPA (tissue Plasminogen Activator, 1 pmol). uPA (0.2 pmol). Pn (plasmin, 100 pmol). MSC sn (MSC supernatant). SDS-PAGE (sodium dodecyl polyacrilamide electrophoresis). **(B)** *Reverse fibrin zymography of MSCs culture supernatants.* MSCs are shown to express active plasminogen activator inhibitor (PAI-1) identified by its molecular mobility compared to references. The Triton X100 gel was overlaid on plasminogen-fibrin-agar plate-gels containing urokinase plasminogen activator (uPA) to reveal the inhibitory activity of PAI-1. kDa (molecular weight position of protein markers in the SDS-PAGE gel). r-PAI-1 (recombinant plasminogen activator inhibitor type 1, 42 pmol). Medium (cell culture medium). MSC sn 122 (cell supernatant 122). MSC sn 92 (cell supernatant 92). uPA (0.2 pmol). The position of purified control references is indicated on the left.

**Figure 2:**
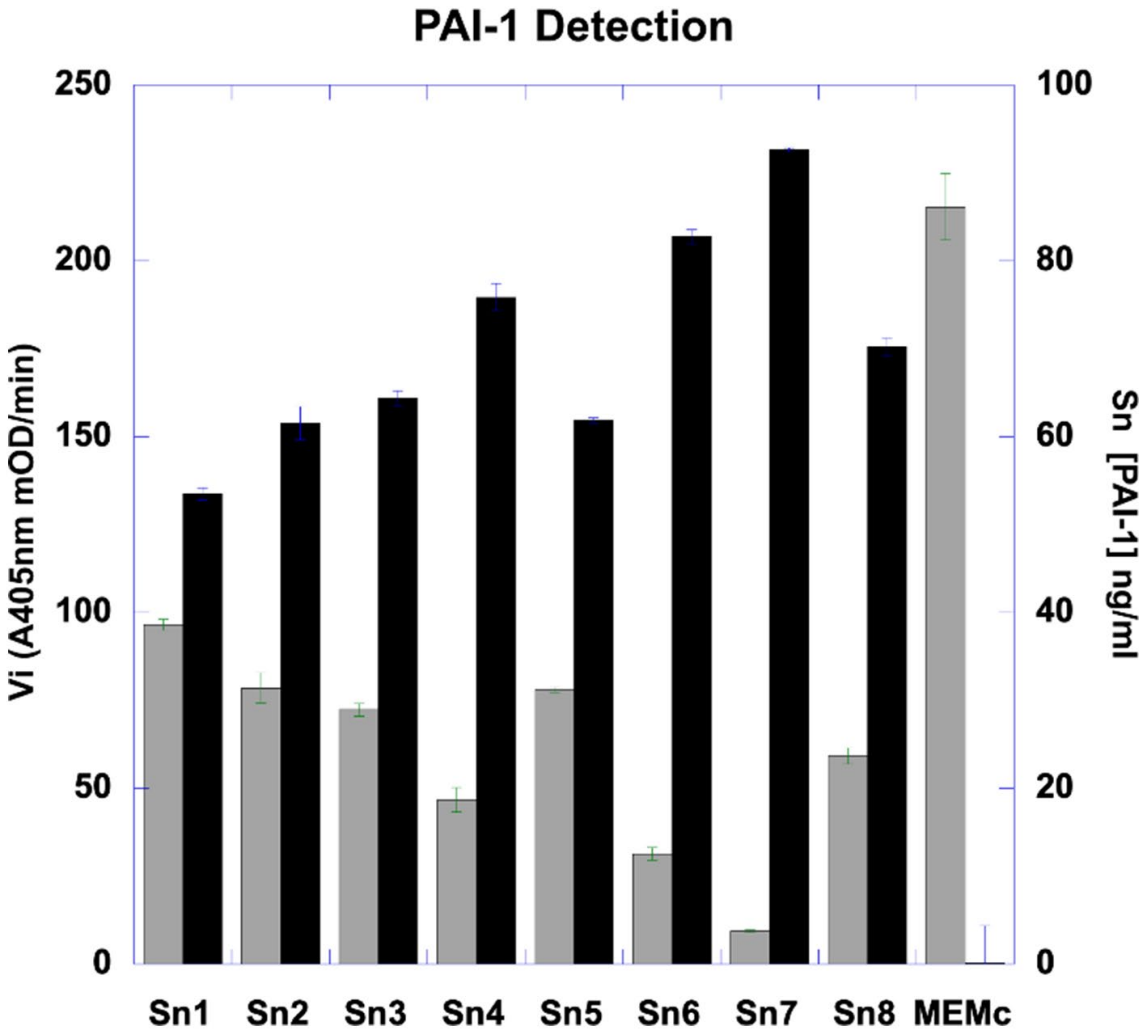
Bar representation of quantification of PAI-1 (ng/mL) expressed by MSC in culture. The concentration of PAI-1 in MSC-conditioned culture medium was quantified by titration with known concentrations of uPA using multi-well plates. The residual-non inhibited uPA was quantified by measuring the amount of plasmin generated from plasminogen using a chromogenic substrate detected by photometry. The amount of uPA inhibited is equivalent to the quantity of PAI-1 present in the supernatant. The graph represents the initial velocity (Vi, left Ordinate) of plasmin generated by residual-non inhibited uPA, expressed in mOD/min detected at a wavelength of A405nm (Gray bars). The corresponding concentrations of PAI-1 (right Ordinate) obtained in different SMC-conditioned culture media (Abscissa) Sn1 to Sn8 (SMS-conditioned culture media) (Black bars). MEMc (minimal modified Eagle culture medium). The PAI-1 concentrations in the supernatants were calculated from the amount of uPA inhibited as indicated in Methods.

Using fibrin zymography we demonstrate that MSCs express uPA as indicated by the position of a fibrinolytic band similar to the migration of purified uPA (Figure 1A). Confirmation was obtained by inhibition of the fibrinolytic activity with specific antibodies. Using reverse fibrin zymography we show that PAI-1 is expressed by MSCs and released in the culture medium (Figure 1B). The amount of PAI-1 secreted in the supernatant was quantitated by titration with uPA (see Supplementary Figure 2). The concentration of PAI-1 was given in ng/ml from the residual uPA units as indicated in Methods (1 IU/mL of inhibited uPA corresponds to approximately 8.7 ng/mL of PAI-1) (Figure 2).

### Detection of the urokinase plasminogen activator receptor (uPAR)

**Figure 3:**
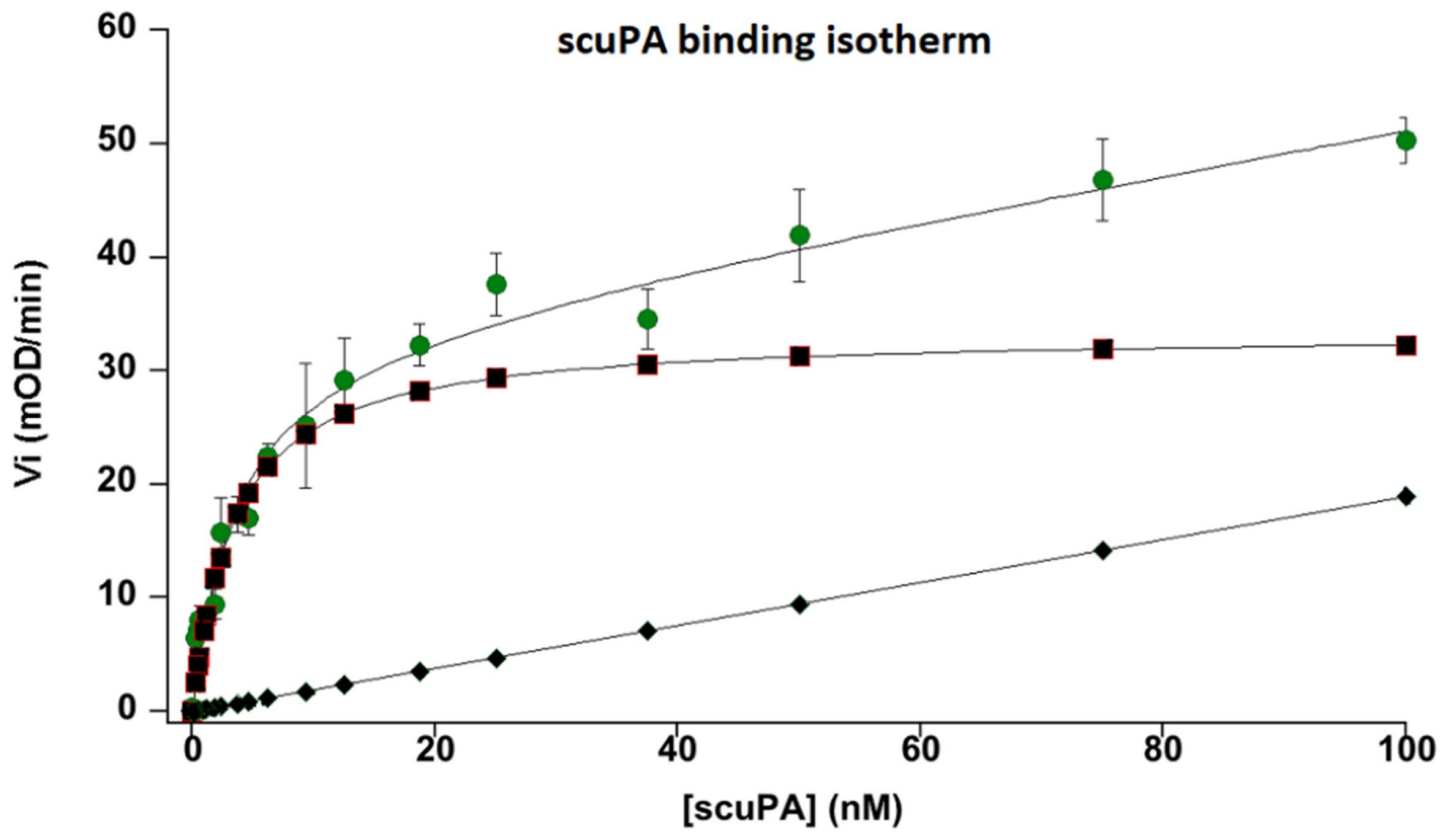
Binding isotherm of single chain urokinase plasminogen activator (scuPA) to its specific MSC urokinase plasminogen activator receptor (uPAR). Different nanomolar (nM) concentrations of scuPA were incubated with MSC at 85% confluence to promote scuPA binding to uPAR. After washing the free not-bound scuPA, plasminogen and a plasmin-selective chromogenic substrate were added at constant concentration to measure the amount of scuPA•uPAR complexes by its capacity to generated plasmin from plasminogen bound to its MSC membrane receptors. The graph represents the initial velocities (Vi, mOD/min) of plasmin generated at the cell membrane by different concentration of bound scuPA. The graph shows a dose-dependent, saturable and specific binding of scuPA to uPAR. An affinity constant, kd = 4 nM was calculated using the Langmuir equation for single-site binding. The dots represent total binding. The lozenges represent non-specific binding. Squares represent specific binding (Total minus non-specific binding).

The uPA receptor, uPAR, was identified at the membrane of MSCs by measuring its capacity to bind scuPA or uPA. Isotherms of the binding were obtained by plotting the plasmin generated using 1 μM plasminogen against the concentration of added scuPA/uPA (Figure 3). The affinity of the interaction, Kd = 4 nM, was calculated using the Langmuir equation for single site binding using Kaleidagraph v.4.5.

**Figure 4:**
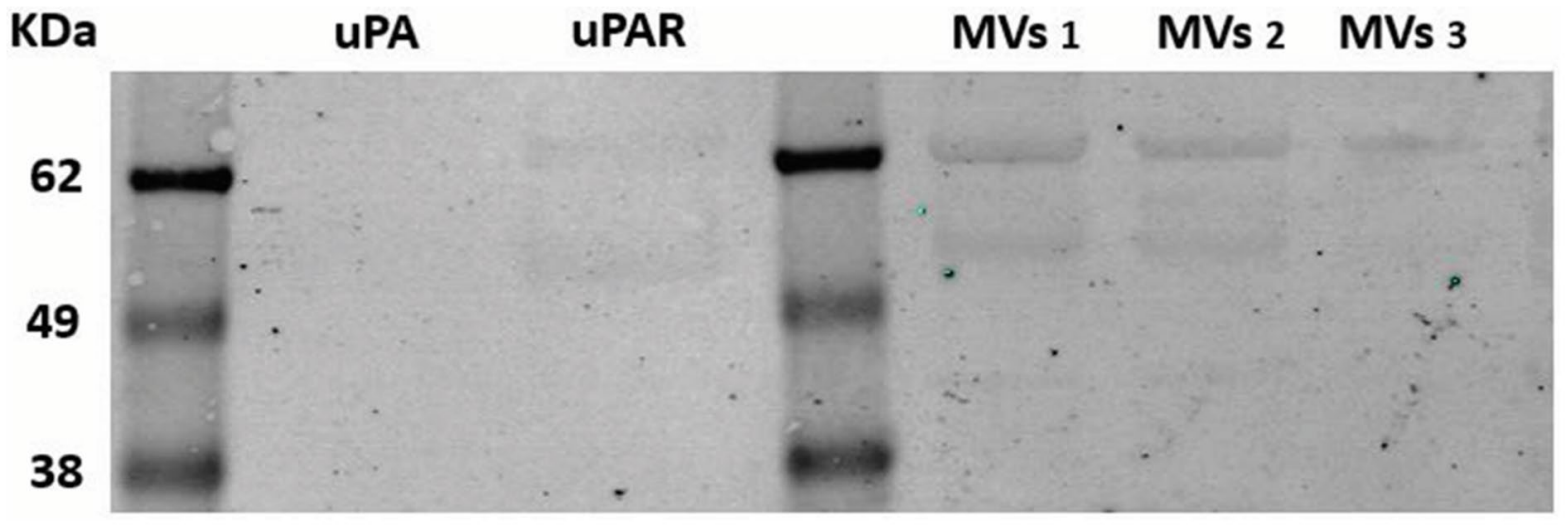
Western blotting analysis of uPAR on MSC-derived microvesicles. Microvesicles were isolated from three different MSCs culture supernatants selected from routine sampling of MSC cultures, as indicated in Methods. Samples were electrophoresed in 10% polyacrylamide gels, blotted on nitrocellulose membrane, incubated with specific anti-uPAR polyclonal antibodies and a secondary anti-rabbit antibody. The membrane was analyzed using a fluorescence Odyseey imager system (LicorBio GmbH, Homburg). Known molecular weight markers are represented at the left. kDA 62, 49, 38. MVs (microvesicles samples 1–3).

The presence of the uPAR protein at the membrane of MSCs or shed MVs was revealed by Western blotting and immuno-detection MVs (Figure 4). The Western blot in Figure 4 shows two bands corresponding to intact uPAR and degraded uPAR in both the purified uPAR and the MVs samples tested.

### MSC activation by plasmin generated at their membrane

**Figure 5:**
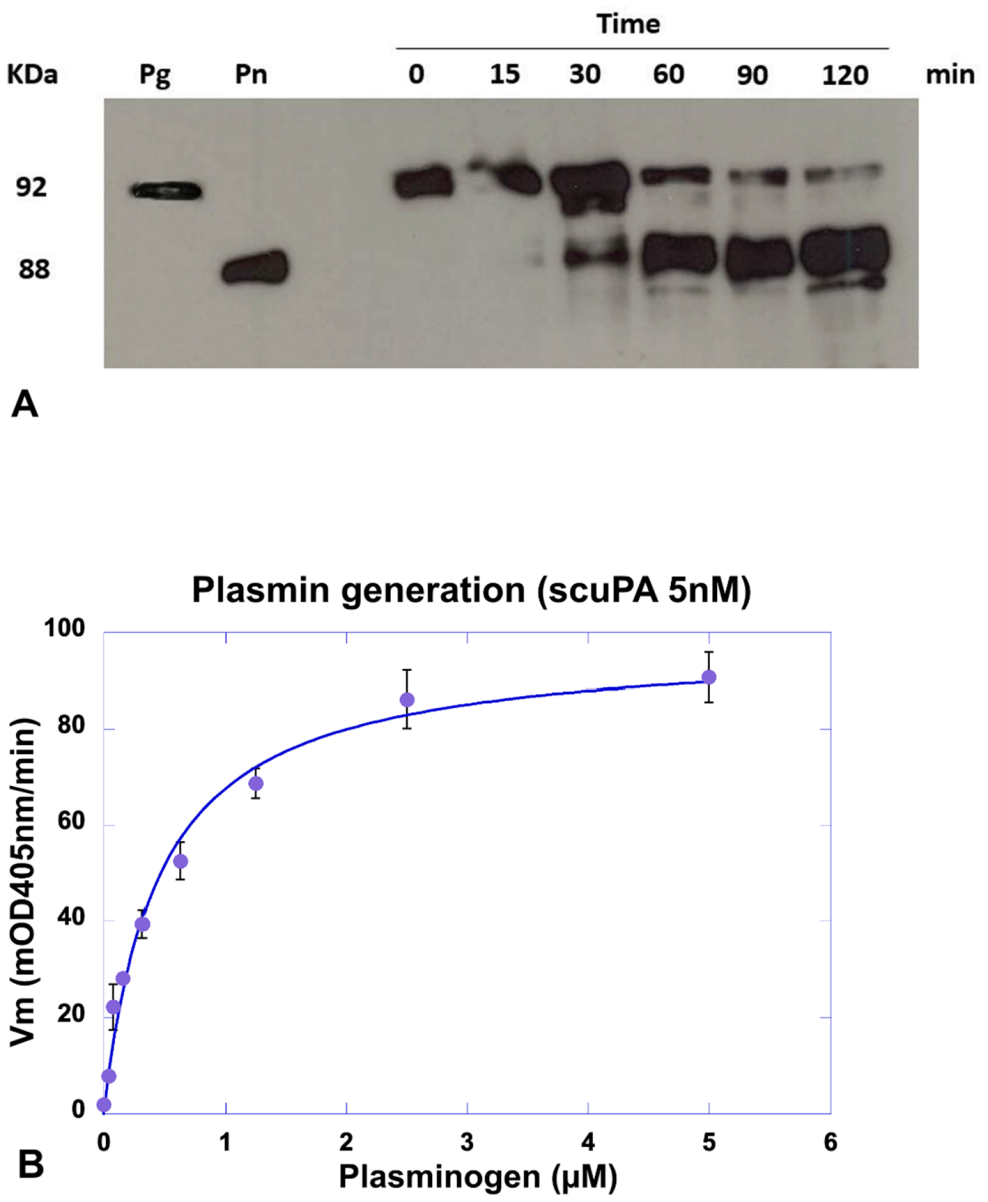
Detection of plasminogen cleavage and plasmin formation at the MSC membrane. MSC cultured in 96-well plates were incubated with a concentration of scuPA equivalent to the Kd (5 nM), after washing plasmin generation was detect by Western blot or plasmin generation kinetics. **(A)** Western blot analysis of membrane lysates. Plasmin generation was detected at different time points (0–120 min) using a constant concentration of plasminogen (1 μM). The supernatant was collected and cells in the wells were lysed for further analysis. The molecular mass of plasminogen (Pg) and plasmin. (Pn) are represented at the left. **(B)** Kinetics of plasmin generation using varying plasminogen concentrations (0–2 μM) and 0.75 mM of the plasmin selective chromogenic substrate CBS0065. Kinetics of plasmin formation was followed during 4 h by measuring the release of *p*-nitroaniline. Data were fitted using the Michaelis–Menten equation and a Km = 0.5 μM was calculated.

Plasminogen incubated with MSCs was assembled on the cell membrane and specifically transformed into plasmin in a time-dependent manner as shown in Figure 5. The amount of plasminogen bound to the membrane decreased as a function of its transformation into plasmin as shown on the Western blot (Figure 5A). Kinetics of plasmin formation by MSC was lysine- and dose-dependent until saturation. The Michalis-Menten constant value calculated from the collected data was Km = 0.5 μM (Figure 5B), is a direct measure of the affinity of plasminogen for its membrane receptor and its transformation into plasmin. Lysine-dependent specific plasminogen binding was indicated by inhibition of plasmin formation both with the lysine analogue TXA that blocks the LBS of plasminogen, and with CpB that cleaves C-ter lysine residues from the cell surface. The generated plasmin was also efficiently inhibited by the plasmin inhibitor, aprotinin.

In contrast, active uPA bound directly to the membrane or resulting from plasmin cleavage of membrane-bound scuPA was protected from inhibition by PAI-1 secreted into the medium by MSCs (see Supplementary Figure 3). However, active uPA added to the conditioned MSC medium was nevertheless complexed by PAI-1, a condition that was used to quantify PAI-1 as indicated above.

### Plasmin generation on MSCs is associated to the release of microvesicles

**Figure 6:**
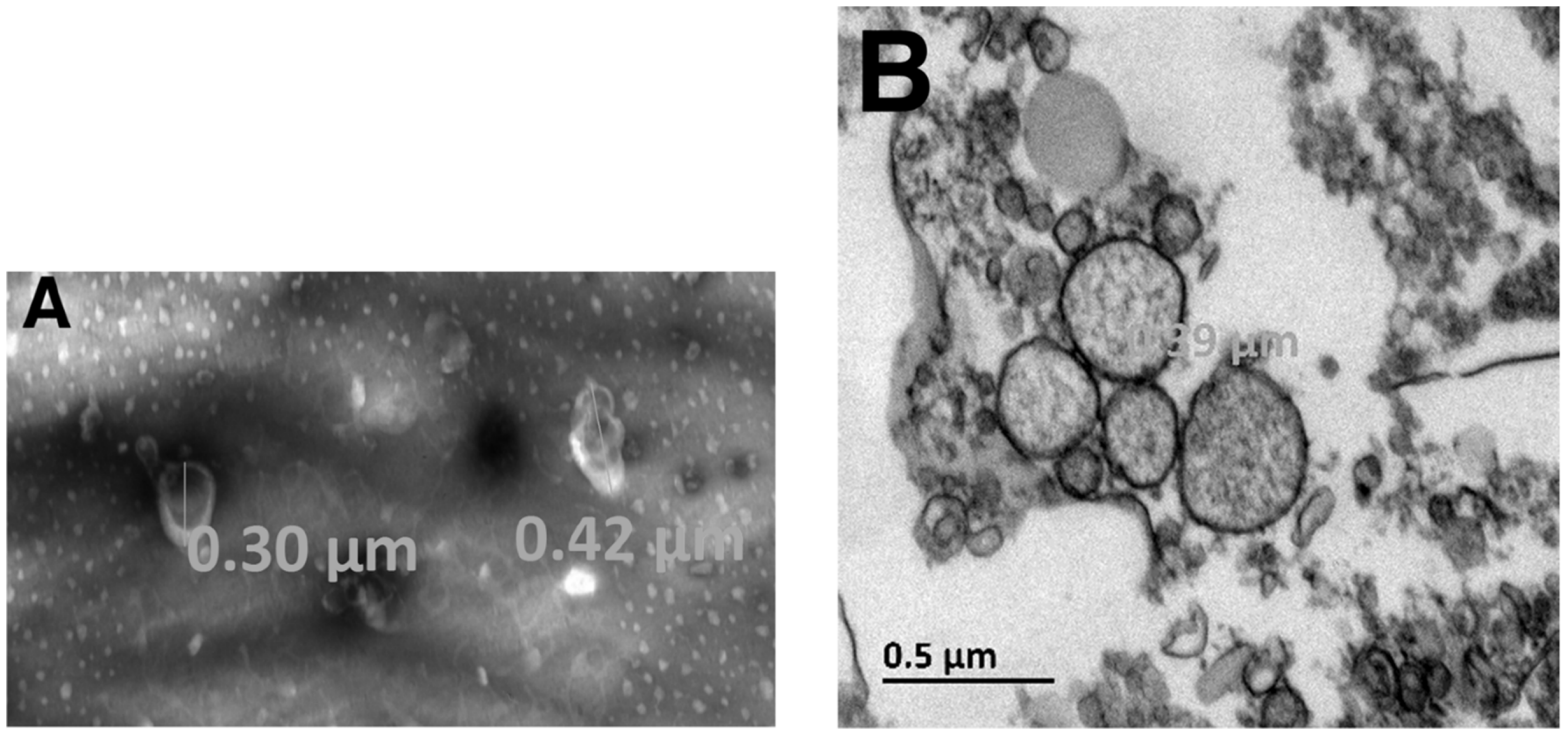
Electron microscopy of MSC-derived microvesicles. Experiments and analysis of electron microscopy images were performed as indicated in Supplementary material. The isolated MVs were defined by their size using negative stain **(A)** and transmission electron microscopy **(B)**. The largest diameter of the microvesicles observed was 390 nm.

We first explored the possibility that MSCs may release MVs under normal culture conditions without added stimulators such as thrombin or TNF-α. The MVs were isolated from the culture medium and their concentration was assessed by measuring total protein content or exposed phosphatidylserine. The amount of MVs per cell number produced by different preparations of MSCs under these conditions is presented in Tables 1, 2. The isolated MVs were defined by their size using negative stain and transmission electron microscopy (Figure 6) (see Supplementary material for EM experimental procedure). Electron microscopy measurements indicate that these MVs are around 300 nm in size and, are surrounded by a well-defined membrane bilayer, contain substructures that have an electron density similar to that of cell cytoplasm. Of note, the use of the indicated stimulators did not increase the number of MVs (not shown), thus suggesting that MSCs in culture release microvesicles at baseline without any external stimulus. However, the small amounts of scuPA expressed by these cells (see Figure 1A) are able to generate plasmin as detected by photometry. Plasmin has indeed been shown to provoke the release of MVs by adherent cells (23, 24). It is therefore possible that MSCs were stimulated by the discrete amount of plasmin directly generated at their membrane from fetal calf serum plasminogen present in the culture medium. As a matter of fact, when resting cells were incubated with a high concentration of plasminogen (500 nM), we observed morphological changes in MSC phenotype (see Supplementary Figure 5).

**Table 1:** Microvesicles quantified by protein content.

Sample	Number of cells	MVs (mg/mL)	MVs (ug)/(10^6^ cells)
1	1.60 × 10^9^	1.82	6.38
2	1.60 × 10^9^	2.03	5.21
3	1.13 × 10^8^	1.74	126.19
4	2.28 × 10^8^	2.00	94.56
5	6.00 × 10^8^	1.00	13.67
6	8.71 × 10^8^	2.22	30.37

Samples obtained from MSCs conditioned medium at different cell confluence. Cell number was determined at 100% (1, 2, 6), 90% (4, 5) and 50% (3) confluence. Cells were counted using a hemocytometer (Malassez) and trypan blue. MVs amount was determined by measuring their total protein content as indicated in Methods.

**Table 2:** Microvesicles quantified by prothrombinase assay and presented as nM phosphatidylserine (Phtdser) equivalent.

Sample	Number of cells	MVs protein (mg/mL)	P MVs Phtdser (nM)
7	1.6 × 10^9^	1.46	740
8	1.6 × 10^9^	1.38	618
9	1.6 × 10^9^	1.53	687
10	1.6 × 10^9^	1.17	513
11	32 × 10^6^	0.93	495
12	32 × 10^6^	0.82	845

Samples obtained from MSCs conditioned medium at different cell number. Number of cells were determined at 90% confluence. MVs amount was measured by both protein and phosphatidylserine content. Phtdser: phosphatidylserine equivalent reported in nanomol/L (nM) by reference to a purified Phtdser calibrator.

### Effects of membrane-bound plasmin on cell phenotype and survival

**Figure 7:**
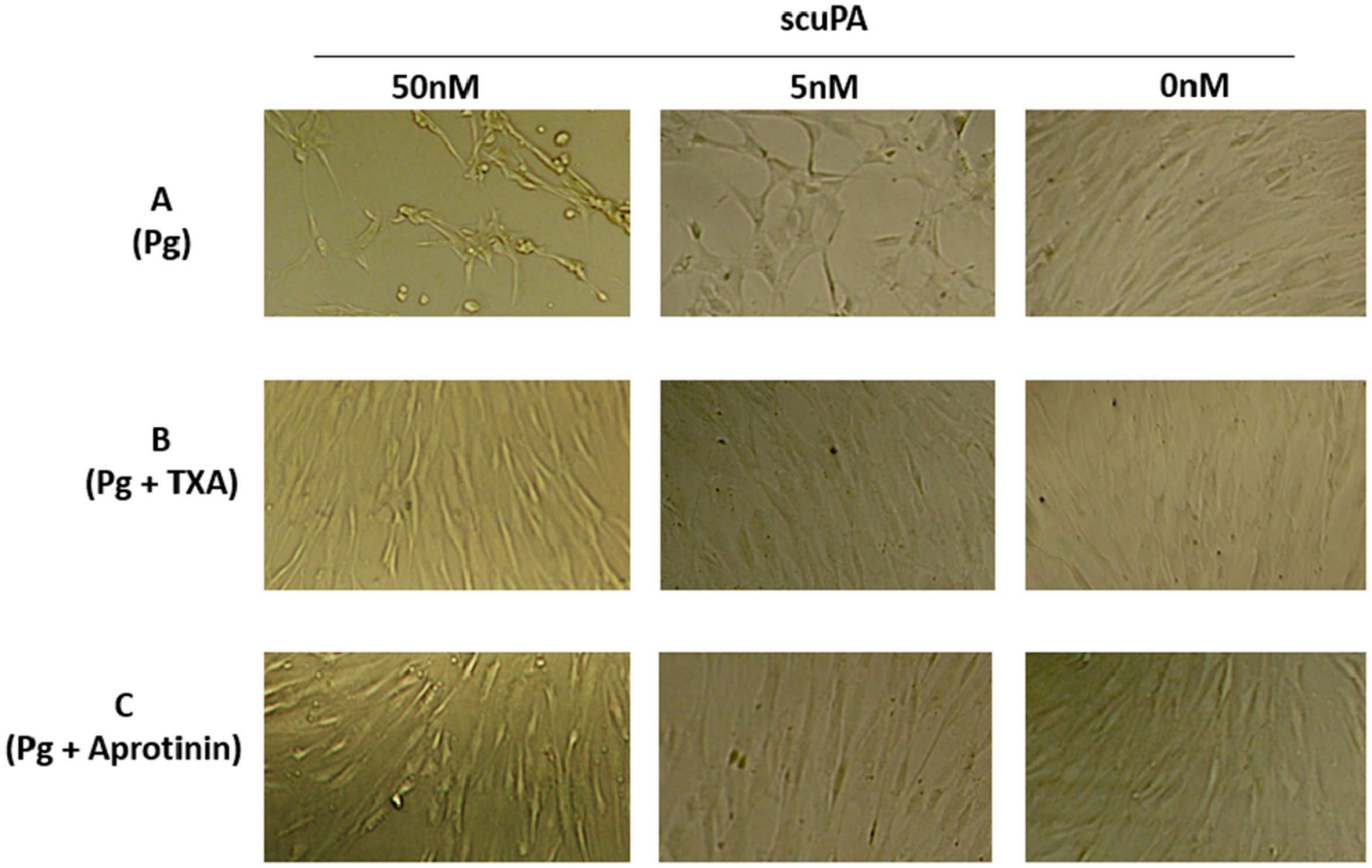
Effect of membrane-bound plasmin generated by scuPA on MSC phenotype. Plasmin was generated at the membrane MSC as indicated in Figure 5 using 0, 5 and 50 nM scuPA and **(A)** Plasminogen at 1 μM. **(B)** Plasminogen 1 μM + TXA 5 mM. **(C)** Plasminogen 1 μM + 11 μM Aprotinin. The micrographs were taken at 40× magnification after 17 h of incubation with the indicated concentrations of plasminogen, TXA and aprotinin.

Besides microvesiculation, plasmin continuously generated at the MSCs is associated with changes in cell phenotype and behavior leading to a decreased amount of residual living cells (MTT assay, not shown). We have previously demonstrated that these changes are related to the proteolytic activity of plasmin on matrix proteins (fibronectin and laminin) (24–26). In the absence of substratum for anchorage, the cells retract and gradually detached from their support. After 24 h of incubation with plasminogen, most MSCs have detached (Figure 7). To investigate the relation between plasmin formation and cell detachment, we used aprotinin (a plasmin inhibitor) and TXA, a lysine-analogue that blocks binding of plasminogen to lysine residues of membrane glycoproteins. As a consequence, plasminogen is unable to co-localize with the uPA•uPAR complex. In the absence of plasmin, the matrix proteins are not degraded, the cells do not detach from their support and remain viable (Figure 7).

### Exogenous regulation of pericellular proteolysis by TXA

**Figure 8:**
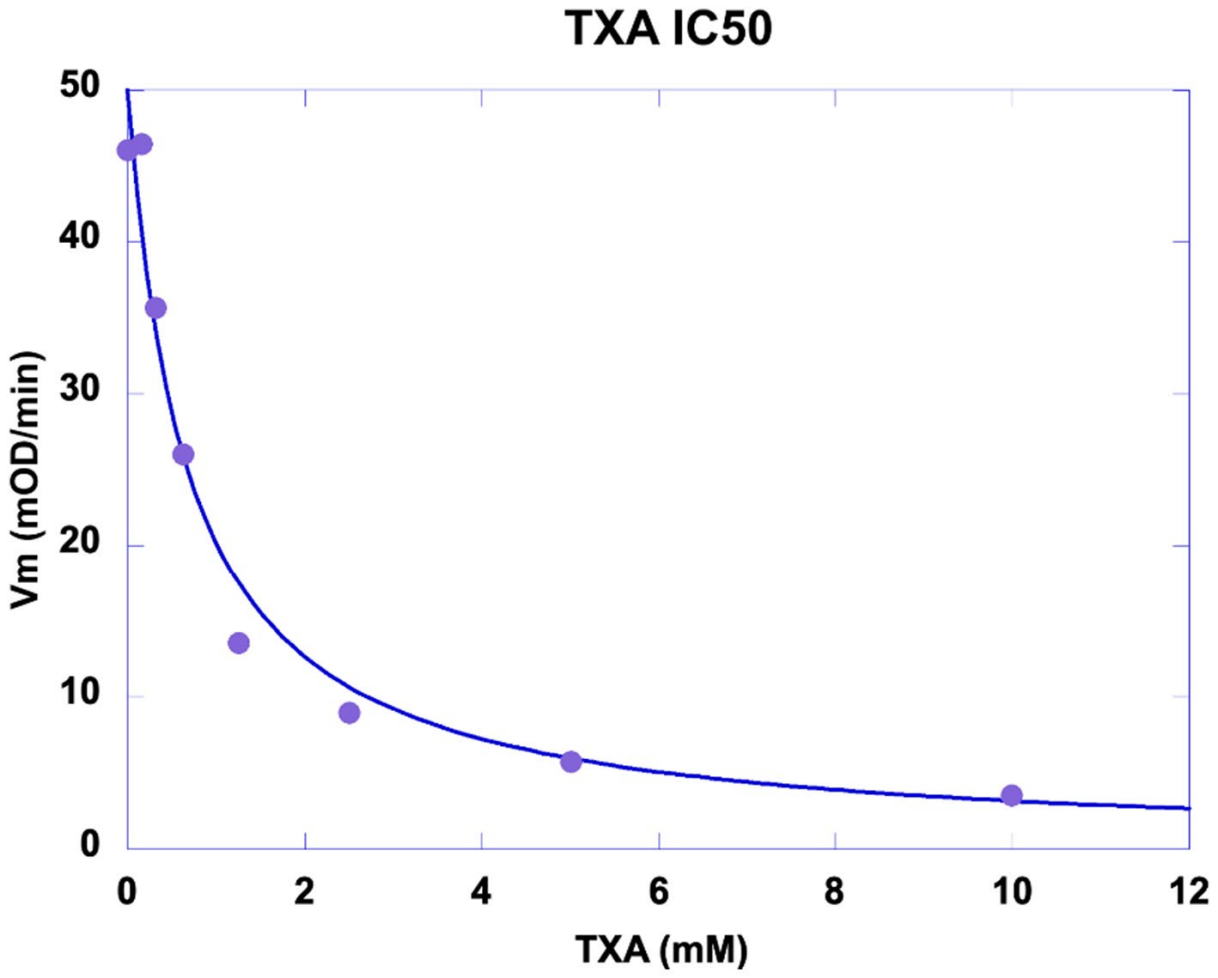
Inhibition of plasmin generation by TXA. MSC were washed and incubated with 5 nM scuPA. Un-bound scuPA was discarded by washing the MSCs which were then incubated with a fixed concentration of plasminogen (500 nM) challenged with 0–10 mM TXA. The graph represents the initial velocity (Vi) of plasmin formation (mOD/min) plotted against the concentrations of TXA. Half-maximal inhibitory concentration (IC50) of Tranexamic acid (TXA) was calculated IC50 = 0.676 mM.

**Figure 9:**
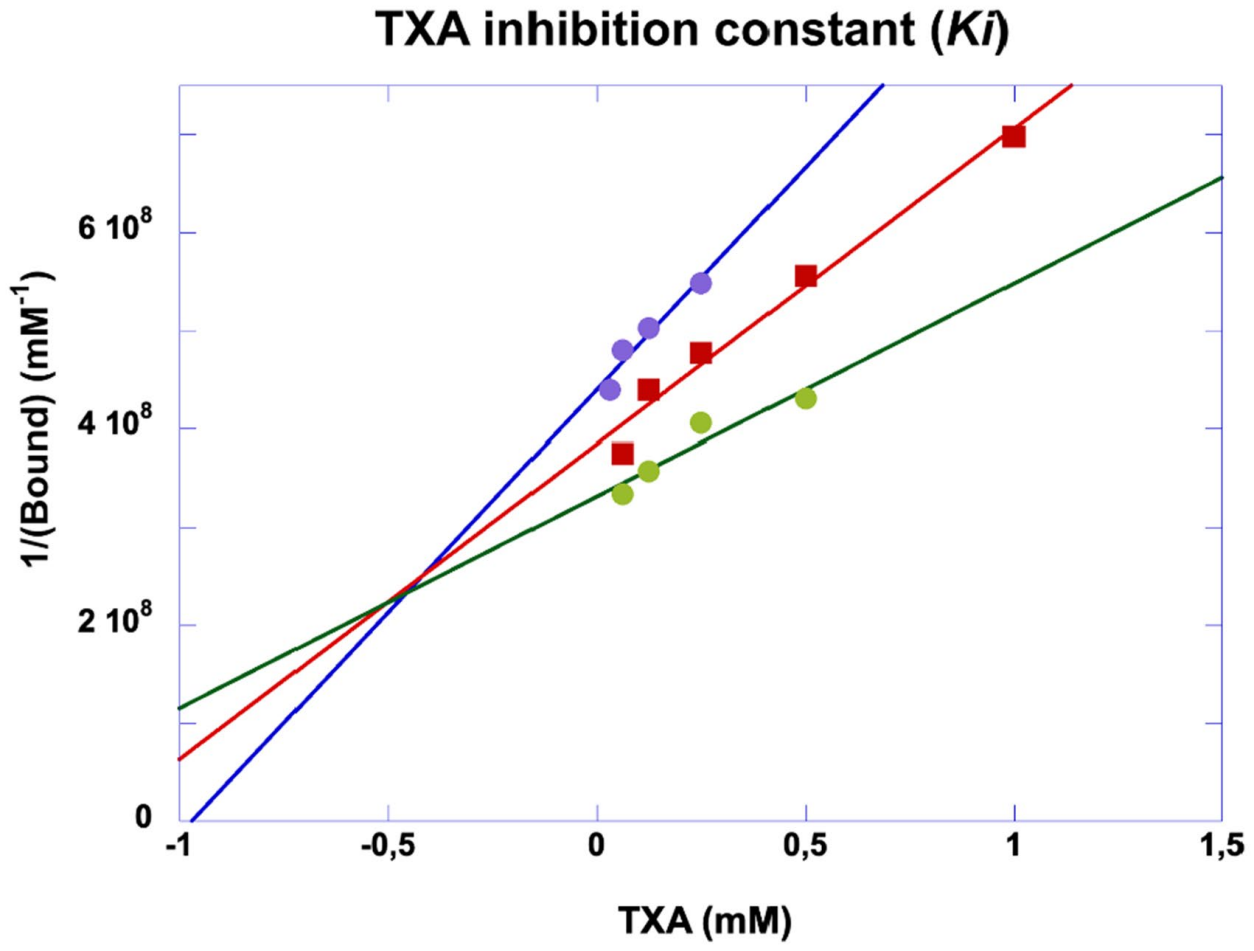
Dixon plot of inhibitory kinetics of tranexamic acid. Experiments were performed as indicated in Figure 5. The inhibition constant (Ki) was obtained by plotting 1/generated plasmin against varying concentrations of tranexamic acid incubated with plasminogen at 200 (purple dots), 300 (red squares) and 400 nM (green dots). The plasmin selective chromogenic substrate was 0.75 mM. The concentration of plasmin was calculated from the initial velocities and the extinction coefficient of the chromogenic substrate. Ki ≈ 0.5 mM.

TXA was shown to be a competitive inhibitor of plasminogen binding to cell membrane glycoproteins. We calculated a half-maximal inhibitory concentration IC50 = 0.676 mM (Figure 8) at a plasminogen concentration of 500 nM. The inhibition constant of this interaction was Ki = 0.48 mM as calculated by Dixon plot using data obtained with 200, 300 and 400 nM plasminogen (Figure 9). A concentration of TXA 5–10-fold higher (5 mM TXA in Figure 7B) was used to obtain complete inhibition of plasmin formation and yet maintaining MSC adherence and viability.

## Discussion

The primary objective of this study was to elucidate the mechanistic pathway of plasmin generation at the MSCs membrane and assess the regulatory role of TXA as a potential therapeutic intervention. Our findings demonstrate that plasminogen from the cell microenvironment, along with uPA and its receptor uPAR expressed on MSCs, assemble at the membrane to generate plasmin. This study provides the first evidence that plasmin generated *in situ* triggers (I) an early cellular response characterized by membrane blebbing and vesiculation, leading to the release of MVs, and (II) extracellular proteolytic activity on matrix proteins, thereby disrupting cell-matrix interactions and promoting phenotypic changes associated with MSC detachment and migration. The released MVs harbor the plasminogen activation machinery, specifically the uPA•uPAR proteolytic complex, which may contribute to tissue remodeling and wound healing in pathological proteolysis.

Interactions between cell receptors, such as integrins, and extracellular matrix glycoproteins (e.g., fibronectin, laminin, collagen, thrombospondins, and tenascins) provide a scaffold for cell adhesion. These interactions mediate cytoskeletal reorganization, leading to the formation of focal adhesions and intracellular signaling (27, 28), which regulate cell growth, morphology, migration, and anchorage-dependent survival (29). Proteolytic disruption of extracellular matrix-integrin interactions can therefore influence membrane cytoskeletal dynamics, altering cell adhesion and survival during tissue remodeling, wound healing, or pathological proteolysis (30–32).

Consistent with previous studies, localized cytoskeletal decoupling from the membrane leads to bleb formation, exposing Phtdser (33). Furthermore, loss of matrix anchoring triggers focal adhesion reorganization, ultimately resulting in cell detachment (34).

In the absence of other morphological or structural changes, these initial membrane alterations indicate a reversible response to stimulation by stressors. We propose that early events such as membrane blebbing and microvesicle formation occur before anchorage-dependent survival is compromised (13, 26, 35–38). Importantly, these MVs can be readily detected in various biological fluids, including MSC culture supernatants, exudates, or secretions, making their analysis a feasible approach for studying cell responses (39).

In our experiments, MSCs spontaneously released MVs in culture without external stimulation. However, zymography confirmed that these cells express low levels of scuPA, which is sufficient to generate plasmin. Plasmin has been shown to trigger MVs release from adherent cells (23, 24), suggesting that MSC-derived MVs in our study may have been generated as a consequence of by plasmin activity at the cell membrane. The efficacy of plasmin generated at MSCs surface is further supported by the insensitivity of uPAR-bound uPA to inhibition by PAI-1. In contrast, uPA released into the conditioned medium was efficiently inhibited by PAI-1, an effect that was leveraged to quantify PAI-1 concentrations.

MSC-driven proteolysis plays a critical role in physiological tissue remodeling, but excessive pericellular plasmin activity may contribute to pathological extracellular matrix degradation, impairing wound healing and predisposing to hemorrhagic complications. In conditions such as metrorrhagia and postpartum hemorrhage, endometrial MSCs may facilitate excessive proteolysis, disrupting matrix integrity and prolonging bleeding. Given the pivotal role of MSC-mediated plasmin activation in the above bleeding processes or in models where tissue remodeling is key, for instance cell transplantation, controlling pericellular proteolysis emerges as a promising therapeutic strategy. One example of the need for improved control of pericellular proteolysis in cell transplantation is the pancreatic islet transplantation in type I severe diabetes patients (40). The engraftment and the restoration of the graft function into the liver host tissue is dependent on the early intra-islet re-vascularization that will ensure full islet perfusion and endocrine function. Indeed, elevated cytokine concentrations known to occur at the vicinity of the transplanted islets immediately after intraportal injection promote mesenchymal transition of the intra-islet endothelial cells, thereby impairing the restoration of an intra-islet vessel with complete endothelium lining. *In vitro*, the endothelial to mesenchymal transition is associated with an elevated early PLAUR transcription suggesting a potential role of uPAR. MSC-derived extracellular vesicles have been studied as the mediators of the benefits of MSCs in the protection of isolated porcine islets against redox insult, suggesting the feasibility of islet graft preconditioning MSC-derived MVs. Whether such therapeutical strategy would favor the islet revascularization by limiting cytokine-induced endothelial transition awaits confirmation in animal human models mimicking the environment of the grafted islet. Indeed, such experimental settings are key before the assessment of a TXA pre-treatment for the limitation of excessive pericellular proteolysis and intra-islet endothelial loss caused by abnormal mesenchymal transition.

Our findings provide the first demonstration that TXA effectively inhibits MSC-driven plasmin generation by competitively blocking plasminogen binding to the uPA•uPAR complex at the cell membrane (Ki = 0.5 mM). This mechanism prevents uncontrolled proteolysis while preserving MSC function, offering a novel approach to regulating pericellular proteolysis in tissue repair. Notably, there are currently no specific treatments targeting MSC-mediated proteolysis in clinical conditions such as abnormal endometrial bleeding (e.g., menorrhagia, metrorrhagia, postpartum hemorrhage). By characterizing the inhibitory effect of TXA on MSC-driven plasmin activation, our study introduces a potential therapeutic strategy to mitigate excessive bleeding and restore extracellular matrix integrity.

A limitation of this study is the absence of *in vivo* experiments, due to the current unavailability of a suitable mouse model within our research facility. Additionally, notable physiological differences between murine and human menstrual cycles such as, the limited volume of menstrual blood and the nature of the estrous cycle in mice, make it difficult to establish a representative *in vivo* model for heavy menstrual bleeding.

In conclusion, our study demonstrates that the experimental approach we designed effectively captures the sequential cellular responses of MSCs to plasmin generation. We identify (I) MV formation as an early response, reflecting membrane remodeling, and (II) a delayed phase characterized by cell detachment and migration, potentially leading to compromised survival. This mechanistic sequence highlights the role of uPAR-uPA-plasminogen assembly in regulating MSC fate, shifting from minor structural alterations to significant cellular changes.

These findings have significant implications for pathological conditions involving excessive pericellular proteolysis, including inflammatory diseases like atherosclerosis (31) and hemorrhagic disorders such as postpartum hemorrhage and menometrorrhagia, where MSC participation is implicated. Importantly, our results establish TXA as the first identified pharmacological modulator of MSC-driven plasmin generation, providing a foundation for developing novel therapeutic interventions aimed at preventing excessive bleeding while preserving the regenerative potential of MSCs. By offering a targeted approach to regulating pericellular proteolysis, TXA holds promise as a valuable clinical tool in conditions where MSC-mediated matrix remodeling is dysregulated.

## Data Availability

The raw data supporting the conclusions of this article will be made available by the authors without undue reservation.

## References

[1] Hass R Kasper C Bohm S Jacobs R. Different populations and sources of human mesenchymal stem cells (MSC): a comparison of adult and neonatal tissue-derived MSC. Cell Commun Signal. (2011) 9:12. doi: 10.1186/1478-811X-9-12, PMID: 21569606 PMC3117820

[2] Neuss S Schneider RK Tietze L Knuchel R Jahnen-Dechent W. Secretion of fibrinolytic enzymes facilitates human mesenchymal stem cell invasion into fibrin clots. Cells Tissues Organs. (2010) 191:36–46. doi: 10.1159/000215579, PMID: 19390164

[3] Copland IB Lord-Dufour S Cuerquis J Coutu DL Annabi B Wang E. Improved autograft survival of mesenchymal stromal cells by plasminogen activator inhibitor 1 inhibition. Stem Cells. (2009) 27:467–77. doi: 10.1634/stemcells.2008-0520, PMID: 19338064

[4] Agis H Kandler B Fischer MB Watzek G Gruber R. Activated platelets increase fibrinolysis of mesenchymal progenitor cells. J Orthop Res. (2009) 27:972–80. doi: 10.1002/jor.20819, PMID: 19030175

[5] Gutova M Najbauer J Frank RT Kendall SE Gevorgyan A Metz MZ. Urokinase plasminogen activator and urokinase plasminogen activator receptor mediate human stem cell tropism to malignant solid tumors. Stem Cells. (2008) 26:1406–13. doi: 10.1634/stemcells.2008-0141, PMID: 18403751

[6] Alfano D Franco P Stoppelli MP. Modulation of cellular function by the Urokinase receptor signalling: a mechanistic view. Front Cell Dev Biol. (2022) 10:818616. doi: 10.3389/fcell.2022.818616, PMID: 35493073 PMC9045800

[7] Ghajar CM Kachgal S Kniazeva E Mori H Costes SV George SC. Mesenchymal cells stimulate capillary morphogenesis via distinct proteolytic mechanisms. Exp Cell Res. (2010) 316:813–25. doi: 10.1016/j.yexcr.2010.01.013, PMID: 20067788 PMC2845480

[8] Plow EF Doeuvre L Das R. So many plasminogen receptors: why? J Biomed Biotechnol. (2012) 2012:141806:1–6. doi: 10.1155/2012/141806, PMID: 23118495 PMC3479832

[9] Bharadwaj AG Kempster E Waisman DM. The ANXA2/S100A10 complex-regulation of the oncogenic plasminogen receptor. Biomol Ther. (2021) 11:1772. doi: 10.3390/biom11121772, PMID: 34944416 PMC8698604

[10] Ditzig Z Wilson CM Salas J Serve KM. Plasminogen binding and activation at the mesothelial cell surface promotes invasion through a collagen matrix. Int J Mol Sci. (2022) 23:5984. doi: 10.3390/ijms23115984, PMID: 35682663 PMC9180734

[11] Fleury V Angles-Cano E. Characterization of the binding of plasminogen to fibrin surfaces: the role of carboxy-terminal lysines. Biochemistry. (1991) 30:7630–8. doi: 10.1021/bi00244a035, PMID: 1677272

[12] Wiman B Wallen P. Activation of human plasminogen by an insoluble derivative of urokinase. Structural changes of plasminogen in the course of activation to plasmin and demonstration of a possible intermediate compound. Eur J Biochem. (1973) 36:25–31. doi: 10.1111/j.1432-1033.1973.tb02880.x, PMID: 4270055

[13] Meilhac O Ho-Tin-Noe B Houard X Philippe M Michel JB Angles-Cano E. Pericellular plasmin induces smooth muscle cell anoikis. FASEB J. (2003) 17:1301–3. doi: 10.1096/fj.02-0687fje, PMID: 12738809

[14] Montes R Paramo JA Angles-Cano E Rocha E. Development and clinical application of a new ELISA assay to determine plasmin-alpha2-antiplasmin complexes in plasma. Br J Haematol. (1996) 92:979–85. doi: 10.1046/j.1365-2141.1996.416951.x, PMID: 8616097

[15] Luther T Magdolen V Albrecht S Kasper M Riemer C Kessler H. Epitope-mapped monoclonal antibodies as tools for functional and morphological analyses of the human urokinase receptor in tumor tissue. Am J Pathol. (1997) 150:1231–44. 9094980 PMC1858180

[16] Hoyer-Hansen G Pessara U Holm A Pass J Weidle U Dano K. Urokinase-catalysed cleavage of the urokinase receptor requires an intact glycolipid anchor. Biochem J. (2001) 358:673–9. doi: 10.1042/bj3580673, PMID: 11535128 PMC1222101

[17] List K Hoyer-Hansen G Ronne E Dano K Behrendt N. Different mechanisms are involved in the antibody mediated inhibition of ligand binding to the urokinase receptor: a study based on biosensor technology. J Immunol Methods. (1999) 222:125–33. doi: 10.1016/S0022-1759(98)00189-6, PMID: 10022379

[18] Freida D Lecourt S Cras A Vanneaux V Letort G Gidrol X. Human bone marrow mesenchymal stem cells regulate biased DNA segregation in response to cell adhesion asymmetry. Cell Rep. (2013) 5:601–10. doi: 10.1016/j.celrep.2013.09.019, PMID: 24139805

[19] Dominici M Le Blanc K Mueller I Slaper-Cortenbach I Marini F Krause D. Minimal criteria for defining multipotent mesenchymal stromal cells. The International Society for Cellular Therapy position statement. Cytotherapy. (2006) 8:315–7. doi: 10.1080/14653240600855905 16923606

[20] Gaussem P Grailhe P Angles-Cano E. Sodium dodecyl sulfate-induced dissociation of complexes between human tissue plasminogen activator and its specific inhibitor. J Biol Chem. (1993) 268:12150–5. doi: 10.1016/S0021-9258(19)50320-9, PMID: 8505335

[21] Gaussem P Angles-Cano E. The formation of complexes between human plasminogen activator inhibitor-1 (PAI-1) and sodium dodecyl sulfate: possible implication in the functional properties of PAI-1. Biochim Biophys Acta. (1991) 1079:321–9. doi: 10.1016/0167-4838(91)90076-C, PMID: 1911857

[22] Hugel B Zobairi F Freyssinet JM. Measuring circulating cell-derived microparticles. J Thromb Haemost. (2004) 2:1842–51. doi: 10.1111/j.1538-7836.2004.00936.x 15456497

[23] Garraud M Khacef K Vion AC Leconte C Yin M Renard JM. Recombinant tissue plasminogen activator enhances microparticle release from mouse brain-derived endothelial cells through plasmin. J Neurol Sci. (2016) 370:187–95. doi: 10.1016/j.jns.2016.09.026, PMID: 27772757

[24] Doeuvre L Plawinski L Goux D Vivien D Angles-Cano E. Plasmin on adherent cells: from microvesiculation to apoptosis. Biochem J. (2010) 432:365–73. doi: 10.1042/BJ20100561, PMID: 20846121 PMC3124466

[25] Rossignol P Ho-Tin-Noe B Vranckx R Bouton MC Meilhac O Lijnen HR. Protease nexin-1 inhibits plasminogen activation-induced apoptosis of adherent cells. J Biol Chem. (2004) 279:10346–56. doi: 10.1074/jbc.M310964200, PMID: 14699093

[26] Kochtebane N Choqueux C Passefort S Nataf P Messika-Zeitoun D Bartagi A. Plasmin induces apoptosis of aortic valvular myofibroblasts. J Pathol. (2010) 221:37–48. doi: 10.1002/path.2681, PMID: 20186923

[27] ffrench-Constant C Colognato H Franklin RJ. Neuroscience. The mysteries of myelin unwrapped. Science. (2004) 304:688–9. doi: 10.1126/science.1097851, PMID: 15118149

[28] Frisch SM Vuori K Ruoslahti E Chan-Hui PY. Control of adhesion-dependent cell survival by focal adhesion kinase. J Cell Biol. (1996) 134:793–9. doi: 10.1083/jcb.134.3.793, PMID: 8707856 PMC2120934

[29] Ellis V Murphy G. Cellular strategies for proteolytic targeting during migration and invasion. FEBS Lett. (2001) 506:1–5. doi: 10.1016/S0014-5793(01)02845-9, PMID: 11591360

[30] Frisch SM Screaton RA. Anoikis mechanisms. Curr Opin Cell Biol. (2001) 13:555–62. doi: 10.1016/s0955-0674(00)00251-9, PMID: 11544023

[31] Rossignol P Angles-Cano E Lijnen HR. Plasminogen activator inhibitor-1 impairs plasminogen activation-mediated vascular smooth muscle cell apoptosis. Thromb Haemost. (2006) 96:665–70. doi: 10.1160/th06-06-0321, PMID: 17080225 PMC2237888

[32] Talhouk RS Bissell MJ Werb Z. Coordinated expression of extracellular matrix-degrading proteinases and their inhibitors regulates mammary epithelial function during involution. J Cell Biol. (1992) 118:1271–82. doi: 10.1083/jcb.118.5.1271, PMID: 1512297 PMC2289583

[33] Daleke DL. Regulation of transbilayer plasma membrane phospholipid asymmetry. J Lipid Res. (2003) 44:233–42. doi: 10.1194/jlr.R200019-JLR200, PMID: 12576505

[34] Mitra SK Hanson DA Schlaepfer DD. Focal adhesion kinase: in command and control of cell motility. Nat Rev Mol Cell Biol. (2005) 6:56–68. doi: 10.1038/nrm1549, PMID: 15688067

[35] Horowitz JC Rogers DS Simon RH Sisson TH Thannickal VJ. Plasminogen activation induced pericellular fibronectin proteolysis promotes fibroblast apoptosis. Am J Respir Cell Mol Biol. (2008) 38:78–87. doi: 10.1165/rcmb.2007-0174OC, PMID: 17656680 PMC2176129

[36] Leskinen MJ Lindstedt KA Wang Y Kovanen PT. Mast cell chymase induces smooth muscle cell apoptosis by a mechanism involving fibronectin degradation and disruption of focal adhesions. Arterioscler Thromb Vasc Biol. (2003) 23:238–43. doi: 10.1161/01.ATV.0000051405.68811.4D, PMID: 12588765

[37] Reijerkerk A Mosnier LO Kranenburg O Bouma BN Carmeliet P Drixler T. Amyloid endostatin induces endothelial cell detachment by stimulation of the plasminogen activation system. Mol Cancer Res. (2003) 1:561–8. 12805403

[38] Zhang X Chaudhry A Chintala SK. Inhibition of plasminogen activation protects against ganglion cell loss in a mouse model of retinal damage. Mol Vis. (2003) 9:238–48. 12813409

[39] Dolo V D'Ascenzo S Violini S Pompucci L Festuccia C Ginestra A. Matrix-degrading proteinases are shed in membrane vesicles by ovarian cancer cells in vivo and in vitro. Clin Exp Metastasis. (1999) 17:131–40. doi: 10.1023/A:1006500406240, PMID: 10411105

[40] Mei L Yuwei Y Weiping L Zhiran X Bingzheng F Jibing C. Strategy for clinical setting of co-transplantation of mesenchymal stem cells and pancreatic islets. Cell Transplant. (2024) 33:9636897241259433. doi: 10.1177/09636897241259433, PMID: 38877672 PMC11179456

