# Supplementary material for: Tranexamic acid impairs plasmin generation on human mesenchymal stem cells and derived membrane microvesicles, halting pericellular proteolysis
Source: Front Med (Lausanne). 2025 Jun 30;12:1570395. doi: 10.3389/fmed.2025.1570395 (PMC12256440; doi:10.3389/fmed.2025.1570395)
Supplement: Supplementary file 1 [file Supplementary_file_1.docx]

**Tranexamic acid impairs plasmin generation on human mesenchymal stem cells and derived membrane microvesicles, halting pericellular proteolysis**

Ramy Abou Rjeily^1^, Christina Mrad^1^, Fatiha Z. El-Ghazouani^2^, Florence Toti ^2^, Audrey Cras^3,4^, Eduardo Angles-Cano^1^.

^1^Université Paris Cité, INSERM, Optimisation Thérapeutique en Neuropharmacologie U1144, 75006, Paris, France

^2^INSERM (French National Institute of Health and Medical Research), UMR 1260, Regenerative Nanomedicine, University of Strasbourg, Strasbourg, France

^3^AP-HP, Hôpital Saint-Louis, Unité de Thérapie Cellulaire, Centre d’Investigation Clinique de Biothérapies CBT501, F-75010 Paris, France.

^4^Université Paris Cité, INSERM UMR 1342, F-75010 Paris, France.

**METHODS**

**Electron Microscopy of isolated microvesicles**

Isolated microvesicles were rinsed 3 times in phosphate buffer (0.1M, pH 7.4) before and after post-fixation with 0.5% osmium tetroxide, dehydrated through a graded ethanol series (70% 2x, 95% 3x, 100% 4x) and finally embedded in epon resin. After polymerization, the coverslips were removed and ultrathin sections were stained with 2% uranyl acetate and lead citrate. All observations were performedwith a JEOL 1011 transmission electron microscope. Images were acquired with a megaview III camera (SIS).

**SUPPLEMENTARY FIGURES**


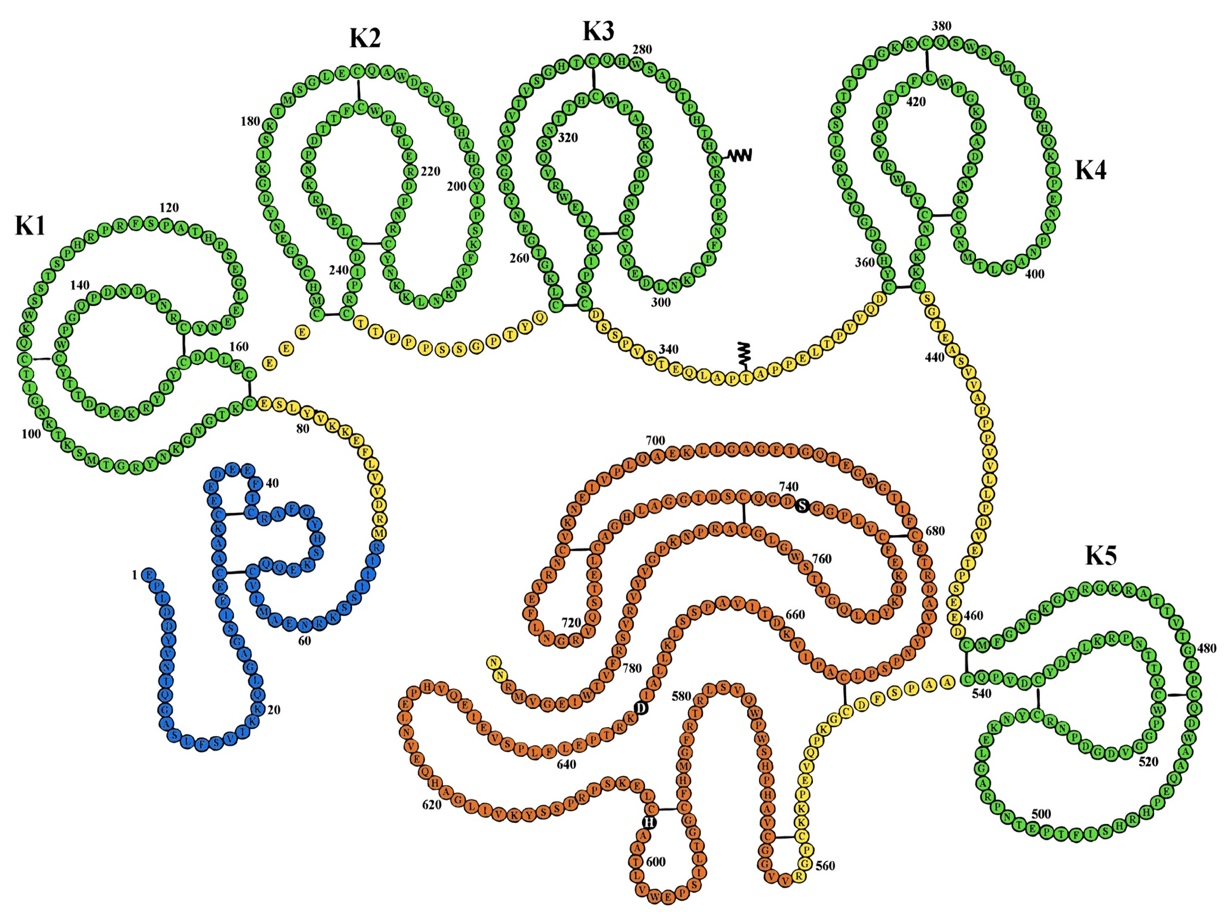


**Supplementary Figure 1. Schematic representation of plasminogen, modified from the plasminogen structure sequence**. (<http://www.chem.cmu.edu/groups/Llinas/res/structure/kringle-big.html>). Full-length plasminogen consists of an N-terminal region, 5 kringle (K) domains and the serine-protease (SP) region. Kringle 1 (K1) and kringle 4 (K4) contain a functional lysine-binding site (LBS). Colour code: Green: K1 to K5, Brown: serine proteinase region. Blue: amino terminal end. Yellow: interconnecting sequences.


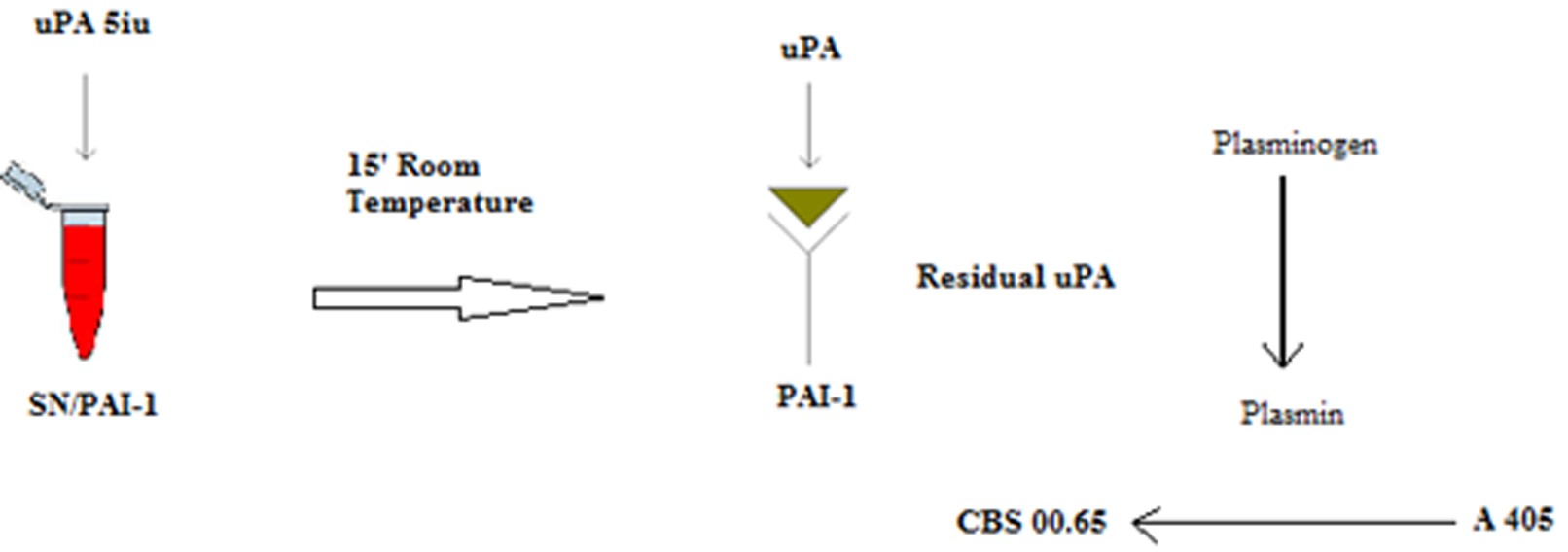


**Supplementary Figure 2: Experimental procedure for the quantification of PAI-1.**

The quantification of PAI-1 in MSC supernatants was performed as indicated in the figure. Briefly equal volumes of supernatant of MSC and 5 IU/ml uPA were incubated for 15 minutes at room temperature. This time was sufficient to ensure PAI-1 present in the supernatant to bind and inhibit the uPA activity. Residual uPA activity was then measured by adding an equal volume of buffer assay (composed of Sodium phosphate 0.05 moles/L, NaCl 0.08 moles/L and Azide 0.01%) containing Plasminogen at 1μM final concentration and chromogenic substrate (CBS) at 0,75 mM final concentration selective for plasmin. The cleavage of the CBS by active plasmin was monitored at 405 nm in a spectrophotometer. The residual uPA activity was calculated using a standard curve constructed by different concentrations of uPA in DMEM. The amount of uPA inhibited is equivalent to the quantity of PAI-1 present in the supernatant that was calculated with the following equation:

**[uPA] _Int_ – [uPA] _Res_ = [uPA] _Inh_ x Dil fact = Quantity of PAI-1 i.u./mL**

Where [uPA]_In_ is the initial concentration of uPA added, [uPA] _Res_ the amount of residual uPA non inhibited by PAI-1 and [uPA]_Inh_ the amount of uPA inhibited by PAI-1 multiplied by the dilution factor of the supernatant. The concentration in i.u/mL was then converted into ng/mL, taking into account the specific activity of uPA and the molecular masse of uPA and PAI-1 to adjust equimolarity.

**Supplementary figures 3 and 4. Inhibition of uPA by PAI-1 at the cellular microenvironment**

The inhibition of uPA by PAI-1 was searched at two levels: Supplementary Figure 3) cell–bound uPA, Supplementary Figure 4) uPA in the surrounding milieu.


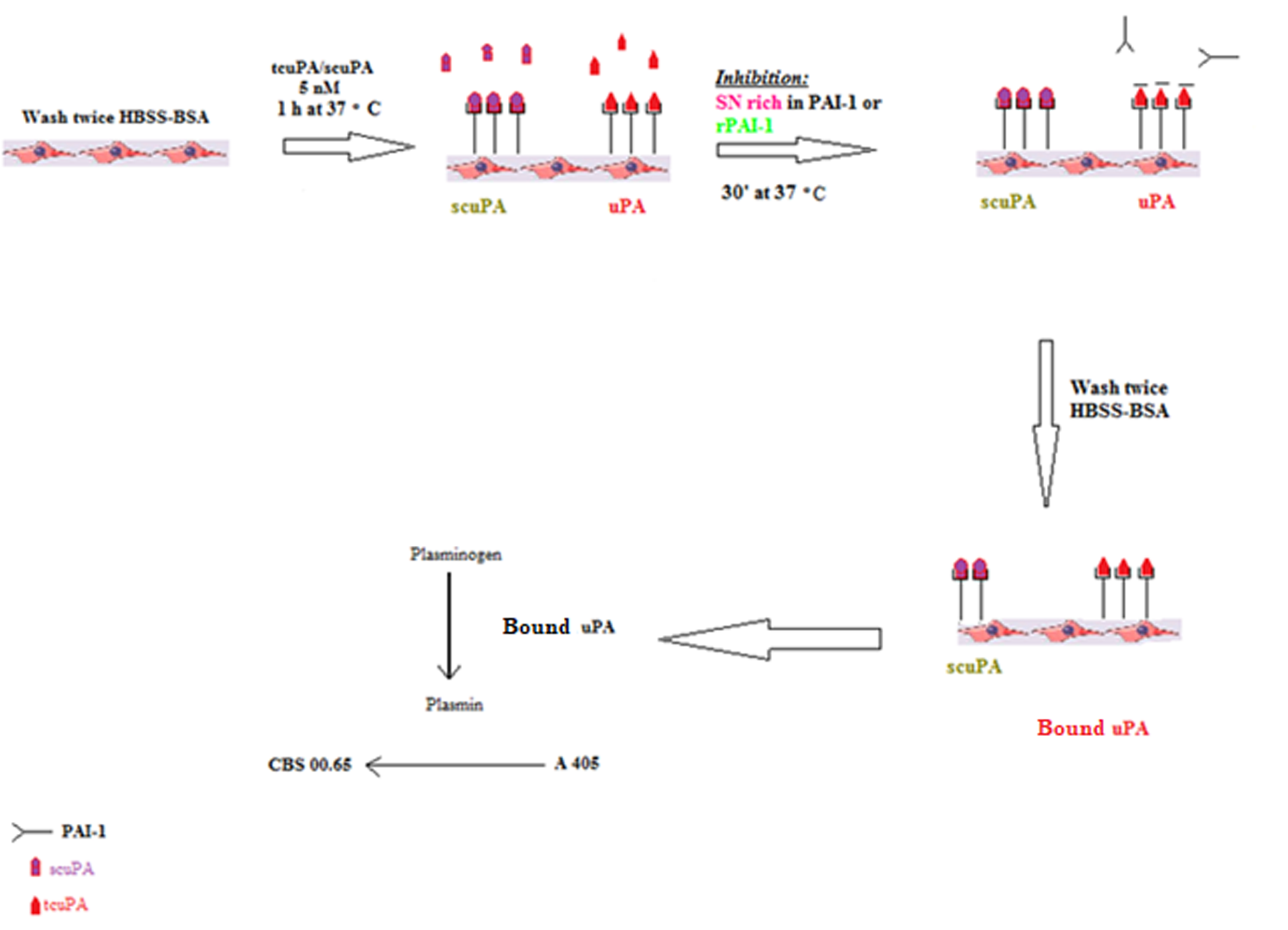


Supplementary Figure 3. Study of the effect of PAI-1 on cell-bound uPA.

Study of the effect of supernatant rich in PAI-1 on cell-bound uPA:

The inhibition of cell-bound uPA by PAI-1 was performed as indicated in Supplementary Figure 3. MSC were washed two times with HBSS-BSA and then incubated with 5 nM of either scuPA or two chain active uPA for 1 hour at 37^◦^C. The cells were washed again with HBSS - BSA and then incubated with a supernatant rich in PAI-1 or with the rPAI-1 for up to 30 minutes at 37^◦^C. After further washings with HBSS-BSA the amount of cell-bound active scuPA or uPA was detected by adding an equal volume of essay buffer containing 1μM plasminogen and 0,75 mM CBS 00.65 final concentrations.


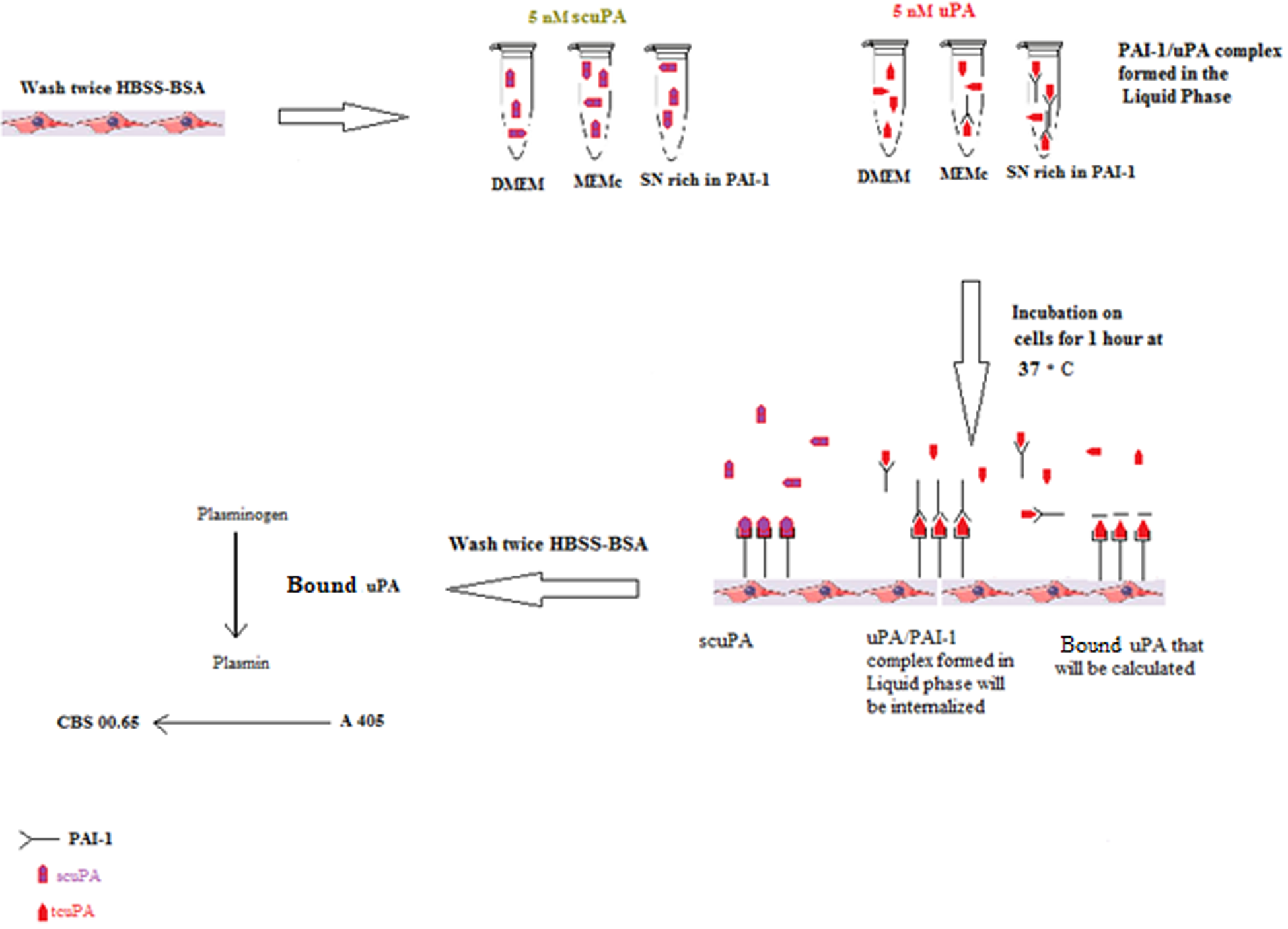


Supplementary Figure 4. The study of the effect of PAI-1 on uPA under conditions mimicking an inflammatory context.

Study of the effect of PAI-1 on uPA added to the cell medium:

In order to mimic the interstitial liquid surrounding the cells where we have an increase in the concentration of PAI-1 and scuPA secreted by the cells, we performed the experiment shown in Supplementary Figure 4. MSC were washed two times with HBSS-BSA and then incubated with a supernatant rich in PAI-1 supplemented with either 5 nM of scuPA or uPA. MEMc, DMEM and rPAI-1 were used as controls. The cells were washed after one hour of incubation at 37 ^◦^C with HBSS-BSA and the amount of cell-bound active scuPA or uPA was detected by adding an equal volume of Assay Buffer containing 1μM plasminogen and CBS 00.65 at 0,75 mM final concentrations.


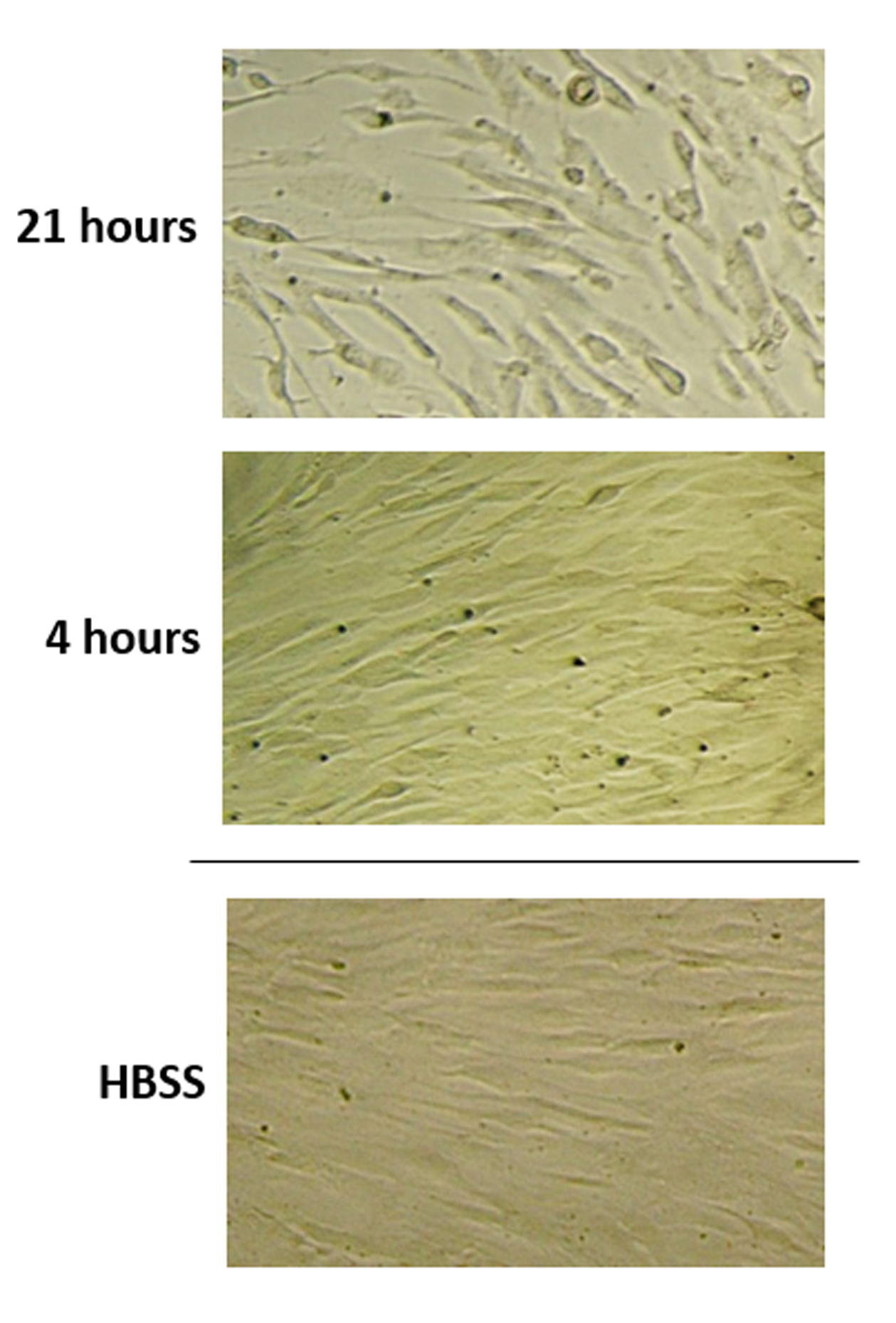


**Supplementary Figure 5. Plasmin effect on MSC phenotype over time**.

MSCs were incubated with 500 nM of plasminogen and no scuPA was added. Morphological changes were compared to control adherent cells, HBSS buffer.

The micrographs were taken at 4 and 21 hours at 40X magnification. The morphological changes observed at 21 hours are related to plasmin formation by scuPA secreted by the cells.
